# Shh and p50/Bcl3 signaling crosstalk drives pathogenesis of BCCs in gorlin syndrome

**DOI:** 10.18632/oncotarget.5103

**Published:** 2015-09-15

**Authors:** Sandeep C. Chaudhary, Xiuwei Tang, Aadithya Arumugam, Changzhao Li, Ritesh K. Srivastava, Zhiping Weng, Jianmin Xu, Xiao Zhang, Arianna L. Kim, Kristopher McKay, Craig A. Elmets, Levy Kopelovich, David R. Bickers, Mohammad Athar

**Affiliations:** ^1^ Department of Dermatology, University of Alabama at Birmingham, Birmingham, AL 35294-0019, USA; ^2^ Department of Biostatistics, University of Alabama at Birmingham, Birmingham, AL 35294-0019, USA; ^3^ Department of Dermatology, College of Physicians & Surgeons, Columbia University, New York, NY 10032, USA; ^4^ Division of Dermatopathology, Department of Dermatology, University of Alabama at Birmingham, Birmingham, AL 35294-4550, USA; ^5^ Department of Medicine, Weill Cornell Medical College, New York, NY 10065, USA; ^6^ Present address: Samuel Oschin Comprehensive Cancer Institute, Los Angeles, CA 90048, USA

**Keywords:** basal cell carcinoma, murine model, NBCCS, Shh, Bcl3

## Abstract

Nevoid basal cell carcinoma syndrome (NBCCS) is a rare autosomal dominant disorder that is due, in large measure, to aberrant Shh signaling driven by mutations in the tumor suppressor gene Ptch1. Here, we describe the development of Ptch1^+/−^/SKH-1 mice as a novel model of this disease. These animals manifest many features of NBCCS, including developmental anomalies and are remarkably sensitive to both ultraviolet (UVB) and ionizing radiation that drive the development of multiple BCCs. Just as in patients with NBCCS, Ptch1^+/−^/SKH-1 also spontaneously develops BCCs and other neoplasms such as rhabdomyomas/rhabdomyosarcomas. Administration of smoothened inhibitors (vismodegib/itraconazole/cyclopamine) or non-steroidal anti-inflammatory drug (sulindac/sulfasalazine) each result in partial resolution of BCCs in these animals. However, combined administration of these agents inhibits the growth of UVB-induced BCCs by >90%. Employing small molecule- and decoy-peptide-based approaches we further affirm that complete remission of BCCs could only be achieved by combined inhibition of p50-NFκB/Bcl3 and Shh signaling. We posit that Ptch1^+/−^/SKH-1 mice are a novel and relevant animal model for NBCCS. Understanding mechanisms that govern genetic predisposition to BCCs should facilitate our ability to identify and treat NBCCS gene carriers, including those at risk for sporadic BCCs while accelerating development of novel therapeutic modalities for these patients.

## INTRODUCTION

Non-melanoma skin cancer (NMSCs) including basal cell carcinomas (BCCs) and squamous cell carcinomas (SCCs) are the most common types of human malignancy [1]. Tumor susceptibility directly correlates with repeated skin exposure to mutagenic solar UVB or environmental chemical carcinogens [2, 3]. UVB induces signature mutations characterized by C to T or CC to TT transitions at pyrimidine-pyrimidine sequences as well as the formation of pyrimidine-pyrimidone photo-products [4, 5]. Highly efficient DNA damage repair mechanisms play an important role in correcting these structural mutations thereby diminishing the risk of tumor development [4]. These mutations are detectable in several tumor suppressor genes particularly Patched 1 (Ptch1) and p53 in sun-exposed skin sites [4–6]. Importantly, 50–80% of sporadic BCCs from both DNA repair-proficient [4, 5] as well as DNA repair-deficient patients with Xeroderma pigmentosum manifest point mutations in both the Ptch1 and p53 genes [7, 8]. Significantly, the characteristic Ptch1 and p53 mutations that occur in sporadic human BCCs are also present in BCCs excised from patients with dominantly inherited Gorlin syndrome also known as nevoid basal cell carcinoma syndrome (NBCCS) [9, 10]. It is now well-established that aberrant sonic hedgehog (Shh) signaling is central to the growth of both sporadic BCCs and those that develop in patients with NBCCS.

NBCCS is a rare autosomal dominant disorder in which affected individuals carry a germline mutation in the Ptch1 gene [11–14]. These patients develop large numbers of BCCs often in the first decade of life as well as medulloblastomas and rhabdomyosarcomas (RMS) [11, 15]. In addition, they are susceptible to numerous benign growths such as epidermal cysts, hair follicle tumors, odontogenic jaw cysts, ovarian cysts as well as characteristic palmar and plantar pits [11, 16, 17]. Multiple skeletal defects also occur including polydactyly, bifid ribs and sprengel deformity. Central nervous system abnormalities may include calcification of the falx cerebri and agenesis of the corpus callosum [18]. Craniofacial abnormalities include frontal bossing and ocular hypertelorism [18].

Family-based linkage studies in affected kindreds led to the identification of the underlying mutation in the Ptch1 gene, a 9q23 microdeletion on the long arm of chromosome 9 [19]. Initially it was thought that mutant Ptch1-based activation of hedgehog (hh) signaling was the sole and pivotal abnormality involved in the pathogenesis of this syndrome [3, 6, 9]. Ptch1 functions as a receptor for hh ligands including Shh, desert (Dhh) and Indian hedgehog (Ihh). Hh is highly conserved and functions as a segmentation polarity gene [6, 20]. During embryogenesis intermittent Hh signaling is a fundamental signal transduction pathway directing the morphogenesis of vital body structures. At birth this pathway is largely silenced throughout adult life [20, 21].

Several animal models of BCCs have been developed based on the identification of Shh signaling abnormalities in these neoplasms [6, 9, 22]. Initial efforts to develop such models focused on Ptch1 null mice which proved to be embryonic lethal. By deleting exons 1 and 2, heterozygous knockout-mice (Ptch1^+/−^) were developed on a C57/BL6 background [23]. Although the skin of these mice appears grossly normal, biopsies show histologic evidence of microscopic basaloid cell proliferation that closely resembles the pattern seen in human BCCs. Skin exposure of Ptch1^+/−^ mice to UVB or to ionizing radiation (IR) induces the growth of microscopic and a few macroscopic BCCs in a pattern more typical of sporadic human BCCs [24–27]. These animals do not manifest the severity of skin involvement that occurs in patients with NBCCS.

Since, the phenotypic features of patients with NBCCS may vary depending upon their ethnic origin [13, 14, 28], we asked whether crossing of Ptch1 heterozygosity into a highly tumor-susceptible genetic background could yield a mouse model that more closely duplicates the NBCCS phenotype. SKH-1 hairless mice have been used for decades as an excellent animal model to study UVB-induced SCCs [29, 30], yet they rarely if ever develop BCCs. Because of the utility of the SKH-1 mouse as a model for UVB-induced SCCs we posited that transfer of Ptch1^+/−^ heterozygosity in C57BL/6 mice onto the SKH-1 background might provide a novel, relevant and more convenient murine model for studying the pathogenesis of BCCs. Importantly, Ptch1^+/−^/C57BL/6 mice or mice of mixed genetic backgrounds rarely develop spontaneous BCCs [25, 27].

In this study, we show that the growth of both spontaneous and UVB-induced BCCs is accelerated in Ptch1^+/−^/SKH-1 mice and that these animals manifest many of the phenotypic abnormalities observed in patients with NBCCS. In addition, similar to NBCCS patients, Ptch1^+/−^/SKH-1 mice are exquisitely sensitive to IR and develop large numbers of BCCs on both their dorsal and ventral skin following exposure to a single dose of 5 Gy. Of further interest in the process of reviewing skin biopsies obtained to assess tumor development in the Ptch1^+/−^/SKH-1 mice we found histologic evidence for a substantially increased pro-inflammatory response, a recognized contributor to the pathogenesis of cutaneous cancers. A significant reduction but not complete abrogation of the growth of BCCs occurs in mice treated with inhibitors of a component of the Shh pathway known as smoothened (SMO) including Erivedge (vismodegib) or Sporanox (itraconazole). Following activation of Shh signaling, SMO is translocated to the tip of the primary cilium, an essential step for driving cell proliferation [31]. This process is inhibited by these drugs thereby blocking Shh signaling both in murine BCCs and in patients with NBCCS [32–35].

In our quest to identify more efficacious mechanism-driven targeted therapy for the multiple BCCs that occur in patients with NBCCS, we have found that pharmacologic inhibition of Shh and inflammatory signaling pathways using combined administration of inhibitors of these signaling pathways leads to almost complete abrogation of BCC growth. The pro-inflammatory pathways involved in the pathogenesis of BCCs includes enhanced expression of cyclo-oxygenase 2 (COX-2), and the non-canonical nuclear factor kappa B (NFκB) signaling pathway regulated by the p50 and Bcl3 heterodimer. Combinatorial inhibition of these signaling pathways together with Smo inhibition led to synergistic regression of BCCs. Our data show that Ptch1^+/−^/SKH-1 mice represent a novel and uniquely relevant model for investigating disease pathogenesis in patients with NBCCS and that maximum abrogation of BCCs requires combined inhibition of Shh and NFkB signaling in these “NBCCS-like” mice. It is our belief that this novel murine model of NBCCS provides unique opportunities for adding to current knowledge regarding the mechanisms that govern genetic predisposition to non-melanoma skin cancer and may also enhance our ability to accelerate development of novel agents for the prevention and treatment of human populations susceptible to BCCs the most common type of human malignancy.

## RESULTS

### Development and characterization of NBCCS murine model

**Figure 1:**
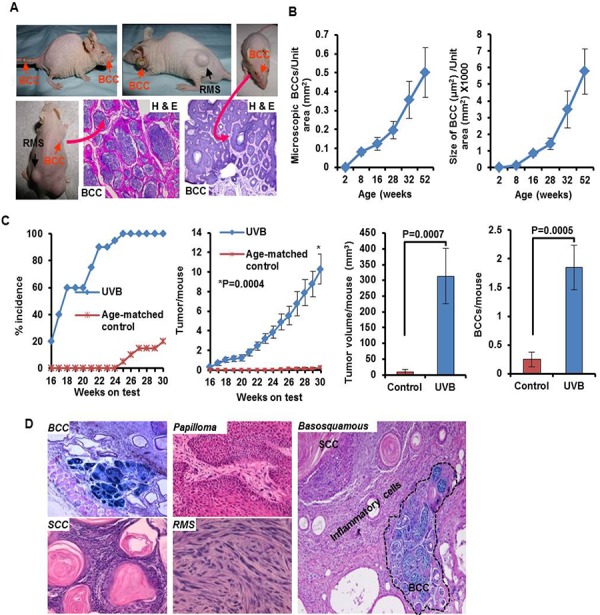
Development of spontaneous and UVB-induced BCCs in Ptch1+/−/SKH-1 hairless mice **A.** Representative pictures of mice showing spontaneous BCCs on nose, dorsal and ventral skin and tail skin, and H&E staining showing histology of these tumors. **B.** Age-dependent progression of microscopic lesions shown as number and size of microscopic BCCs/unit area (mm^2^); **C.** Graphs showing UVB-induced skin tumor development depicted as percentage tumor incidence, tumors/mouse, BCCs/mouse and tumor volume/mouse in Ptch1^+/−^/SKH-1 mice. **D.** Histology of BCCs, papilloma, SCCs, rhabdomyosarcoma and basosquamous carcinoma. Shown in blue is β-gal staining of basaloid lesions.

C57BL/6 mice are generally regarded as tumor-resistant, whereas the SKH-1 mouse offers a tumor-susceptible genetic background for studies of UVB-induced skin photocarcinogenesis [29, 30]. Because of this enhanced skin tumor susceptibility, SKH-1 mice have been extensively used for decades to investigate the pathogenesis of UVB-induced SCCs. They develop numerous SCCs following chronic exposure to UVB in a pattern analogous to that of patients with sequential growth of multiple benign pre-malignant lesions (papillomas) some of which progress to malignant SCCs [30]. However, these mice do not develop BCCs either spontaneously or following chronic UVB irradiation and indeed until recently there was really no useful animal model available for the study of BCCs. Our laboratory has been attempting to develop a mouse model with enhanced susceptibility to cutaneous BCCs. Our strategy employed crossing Ptch1^+/−^/C57BL/6 mice with SKH-1 hairless mice (Supplemental Figure S1A). Animals employed in these studies have been bred for more than 25 generations (Supplemental Figure S1A and S1B). Ptch1^+/−^/SKH-1 hairless mice develop spontaneous BCCs, that appear in areas near the nose, tail and/or randomly on various areas of the dorsal or ventral skin (Figure 1A, upper panel). In contrast, spontaneous development of SCCs in parental SKH-1 or in the Ptch1^+/−^/SKH-1 hairless mice is decidedly uncommon. Significantly, Ptch1^+/−^/SKH-1 hairless mice also manifest rhabdomyomas/rhabdomyosarcomas that generally appeared subcutaneously, predominantly over the hind limbs (Figure 1A, lower panel). These tumors also occur in patients with NBCCS [15] further attesting to the close resemblance of this mouse model to the human disease. In addition, a few of these animals manifest other phenotypic features of NBCCS including polydactyly, craniofacial abnormalties and odontogenic keratocyst(s) (Supplemental figure S2A).

**Figure 2:**
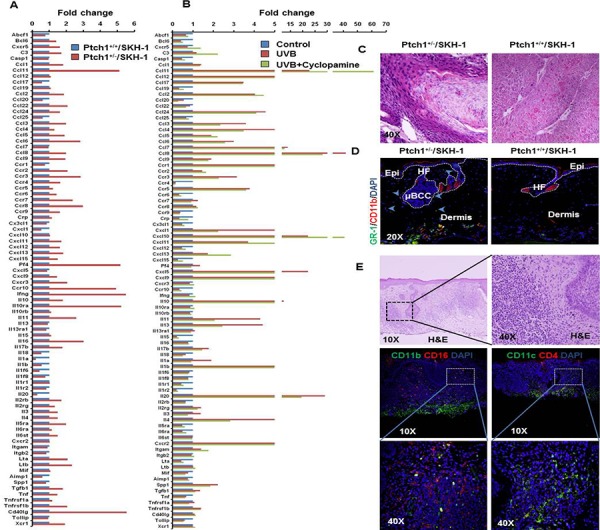
Inflammatory responses are augmented in the skin of Ptch1^+/−^/SKH-1 mice as compared to Ptch1^+/+^/SKH-1 mice and treatment with the SMO inhibitor cyclopamine attenuates these inflammatory responses **A.** Bar diagram showing levels of relative gene expression of inflammatory cytokines/chemokines and their receptors in Ptch1^+/+^/SKH-1 and Ptch1^+/−^/SKH-1 mice. **B.** Bar diagram showing reduction of UVB-induced inflammatory response by cyclopamine in Ptch1^+/−^/SKH-1 mice. **C.** Comparison of pro-inflammatory tumor microenvironment in identical SCCs-like lesions excised from the skin of chronically UVB-irradiated Ptch1^+/−^/SKH-1 and Ptch1^+/+^/SKH-1 mice (original magnification 40X). **D.** Immunofluorescence staining showing presence of GR-1 (green)/CD11b (red)-positive myeloid cells around microscopic BCC-associated tumor stroma of age-matched control of Ptch1^+/−^/SKH-1 and skin of Ptch1^+/+^/SKH-1 mice. HF, hair follicle; Epi, epidermis; μBCC, microscopic BCC (original magnification 20X). **E.** H&E and immunofluorescence staining of the skin of patients with NBCCS showing presence of pro-inflammatory hematopoietic cells. Shown here are CD11b(green)/CD16(red)-positive and CD11c(green)/CD4(red)-positive cells in tumor stroma.

Histopathologic examination of skin tumors in our Ptch1^+/−^/SKH-1 animals revealed interfollicular basaloid cell proliferation in patterns virtually indistinguishable from human BCCs, and trichoblastomas typical of patients with NBCCS (Figure 1A, lower panel and Supplemental Figure S2B and S2C). None of these spontaneous tumors grew in littermates carrying the wild-type Ptch1 gene (data not shown). Spontaneous tumor growth in the Ptch1^+/−^/SKH-1 animals was age-dependent, and usually occurred beyond 18 weeks of age when visible macroscopic BCCs begin to appear (Figure 1B and Supplemental Figure S2D). By 40–50 weeks of age, nearly half the animals were found to have at least one spontaneous visible BCC (Supplemental Figure S2C). More impressive was the age-dependent increase in the number and size of microscopic BCC-like lesions (Supplemental Figure S2D). Between week 8 and week 52 of age there was on average a 4–6-fold increase in microscopic BCCs (Figure 1B). To the best of our knowledge, this is the first murine model manifesting such a high incidence of spontaneous BCCs during the first year of life. Moreover in earlier described murine models, spontaneous BCCs occurred very late in life with a prevalence of less than 10% [6, 27]. In this regard, the Ptch1^+/−^/SKH-1 mouse more closely mimics patients with NBCCS who often develop BCCs in the first decade of life after which the rate of tumor growth typically increases with age [6, 9, 36].

### UVB-induced carcinogenesis in Ptch1^+/−^/SKH-1 mice

The detailed tumor development profile following chronic UVB-irradiation of genetically homogeneous Ptch1^+/−^/SKH-1 mice (25^th^ generation) is shown in Figure 1C. Initial tumors are visible after 14–16 weeks of UVB irradiation (Figure 1C). By week 20, approximately 60% of the animals develop tumors and by week 26, 100% of the mice have at least one tumor (Figure 1C, left panel). By week 30, each mouse had on average, 10.3 ± 1.51 tumors (Figure 1C, middle left panel) of which 1.85 ± 0.39 were BCCs (Figure 1C, right panel). However by week 30, 20% or less of the age-matched control non-irradiated Ptch1^+/−^/SKH-1 mice had developed spontaneous BCCs (0.25 ± 0.12 tumor/mouse). These tumors were also smaller as compared with UVB-induced BCCs (9.5 ± 8.8 vs 312.8 ± 87.8 mm^3^/mouse) (Figure 1C, middle right panel). Immunohistochemical studies of BCCs showed interfollicular basaloid proliferation with strong positive staining for β-galactosidase (β-gal), Gli1, and Hhip (Supplemental Figure S3). To further document the proliferative properties of these BCCs, we immunostained tumor tissue with an antibody specific for keratin-17, an outer root sheath marker protein [37] (Supplemental Figure S3). Keratin-17 expression was consistently increased in these tumors just as it is in human BCCs and those that develop in Gli2 transgenic mice [38, 39]. In addition, upregulation of cyclins D1/D2/D3 and E and augmented expression of Shh signaling-related genes were noted not only in BCCs but also in the tumor-adjacent hyperplastic epidermis (Supplemental Figure S4A).

**Figure 3:**
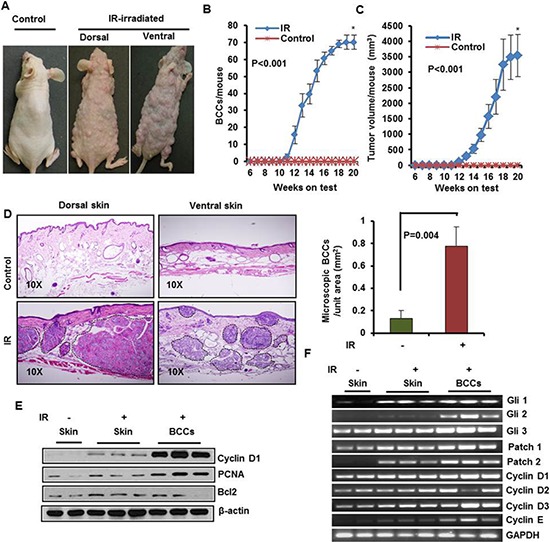
Ionizing radiation (IR) induce multiple BCCs in Ptch1^+/−^/SKH-1 mice **A.** Representative pictures of Ptch^+/−^/SKH-1 mice showing IR-induced visible BCCs on dorsal and ventral skin. **B.** BCCs/mouse. **C.** tumor volume/mouse; **D.** Histology of BCCs from dorsal and ventral skin, and analysis of microscopic BCCs/unit area (mm^2^) in IR-irradiated mice. **E.** Immunoblot analysis of biomarkers predictive of cell proliferation (PCNA and cyclin D1) and anti-apoptotic protein Bcl2 in BCCs of IR-irradiated mice. **F.** transcriptional expression of Glis, Ptchs and cell cycle regulatory cyclins in IR-irradiated BCC. Ptch^+/−^/SKH-1 mice were irradiated with a single dose (5Gy) of IR. The experiment was terminated at week 20 following irradiation. Skin and tumors were excised for histological and for molecular analysis.

**Figure 4:**
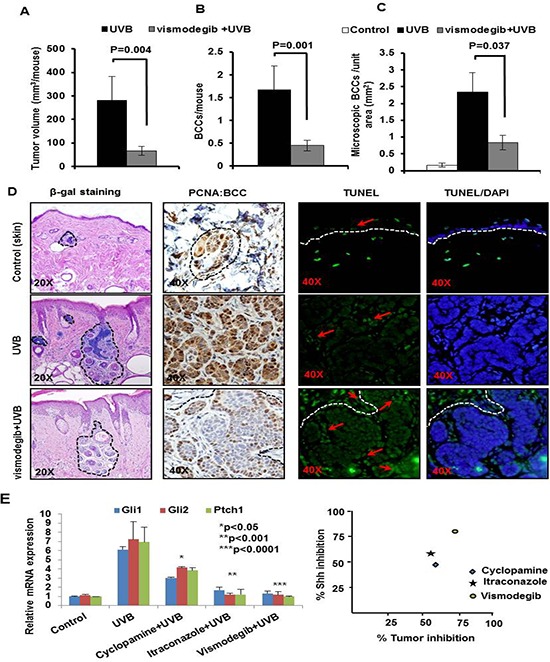
The SMO inhibitor Vismodegib partially attenuates UVB-induced BCC development in Ptch1+/−/SKH-1 mice Ptch1^+/−^/SKH-1 mice were treated with orally administered vismodegib (40 mg/kg body weight twice weekly), 30 min prior to UVB irradiation (180 mJ/cm^2^). Tumor data were recorded each week. Data showing **A.** tumor volume/mouse (mm^3^). **B.** BCCs/mouse. **C.** microscopic BCCs/unit area of skin (mm^2^) of vismodegib- and vehicle-treated in UVB-irradiated Ptch1^+/−^/SKH-1 mice. **D.** β-gal staining of UVB-induced microscopic BCCs (magnification 20x), proliferative biomarker (PCNA) (magnification 40x) and TUNEL staining (magnification 40x) in BCCs from vismodegib- and vehicle-treated mice and **E.** relative ability of vismodegib, ITRA and cyclopamine to inhibit UVB-induced Shh signaling in Ptch1^+/−^/SKH-1 mice and its correlation with BCC growth inhibition.

Importantly, approximately 5% of these animals developed basosquamous carcinoma, an uncommon and typically aggressive human BCC variant that has features of both BCCs and SCCs (Figure 1D, left panel). Indeed these tumors in our mice were highly invasive. Insofar as we are aware no other mouse models have been shown to develop this type of tumor.

Not surprisingly given their SKH-1 background, the chronically UVB-irradiated Ptch1^+/−^/SKH-1 mice also developed squamous papillomas and SCCs. Some of these lesions were poorly differentiated invasive SCCs showing dermal involvement while simultaneously showing signs of squamous differentiation as evidenced by the presence of multiple keratin pearls. These keratotic squamous lesions also resembled invasive keratoacanthomas (Supplemental Figure S4B) and showed dense inflammatory cell infiltrates particularly in the tumor stroma. There were differences in the pattern of tumor-associated inflammation in the Ptch1^+/−^/SKH-1 animals as compared to the parental SKH-1 strain as shown in figure 2C and Supplemental Figure S4B. Comparison of identical UVB-induced SCC-like lesions in Ptch1^+/−^/SKH-1 and Ptch1^+/+^/SKH-1 mice (figure 2C) revealed that the tumors induced in Ptch1^+/−^/SKH-1 mice were more aggressive and showed conversion of highly polarized epithelial cells into mesenchymal-like spindle structures infiltrating into the dermis. As shown in Supplemental Figure S4B, these tumor infiltrates were also associated with inflammatory hematopoietic cells.

### Shh signaling in Ptch1^+/−^/SKH-1 enhances inflammatory microenvironment in the skin

Next we attempted to characterize the tumor microenvironment in the skin and BCCs of Ptch1^+/−^/SKH-1 mice by profiling baseline and UVB-induced expression of cytokines, chemokines; inflammatory cell infiltration; pro-inflammatory signaling; and responses to non-steroidal anti-inflammatory agents.

First, we used the Inflammatory Cytokines & Receptors RT^2^ Profiler PCR Array to profile the expression of 84 key genes known to mediate inflammatory responses. These inflammatory cytokines and chemokines are reported as secretory molecules which augment recruitment of innate immune cells including macrophages, neutrophils, mast cells, myeloid-derived suppressor cells (MDSCs), dendritic cells, and natural killer cells as well as adaptive immune cells (T and B lymphocytes) into the tumor microenvironment [40, 41].

We observed that baseline expression of 49 of 84 genes was higher, 19 were lower and 16 remained unaltered in Ptch1^+/−^/SKH-1 murine skin as compared to Ptch1^+/+^/SKH-1 animals (Figure 2A). Among these, chemokines (Ccl11, Ccl22, Ccl3, Ccl5, Ccl6, Ccl8, Ccl9, Cxcl13 and Tgfb1), chemokine receptors (Ccr2, Ccr3, Ccr7, Ccr8, Ccr10 and Cxcr3), interleukins (IL) (10, 11, 12b and 16), interleukin receptors IlRa (10 and 5) and other cytokines such as Pf4, Ifng, Cd40lg, Lta, Ltb and Tnfrsf1b were up-regulated more than 2-fold. Approximately a 50% reduction in the expression of chemokines (Ccl17, Ccl20, Ccl25 and Cxcl1 and ILs (18, 1a, 1b, 1f6 and 20), and cytokine Aimp1 was seen (Figure 2A and Supplemental Figure S5A).

**Figure 5:**
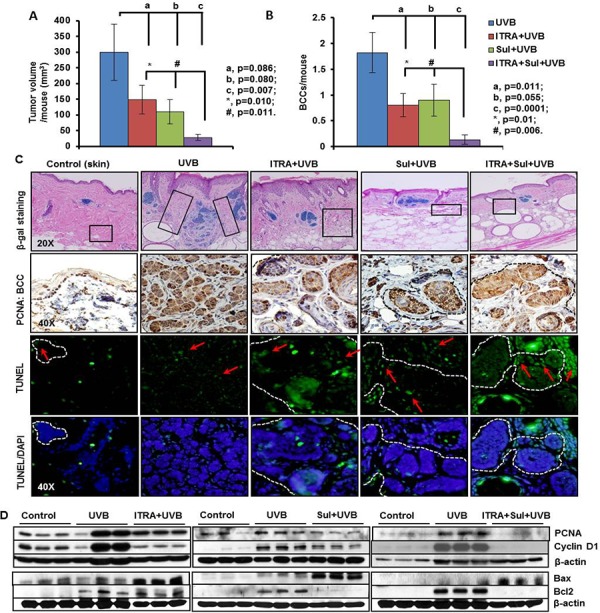
The NSAID Sulindac (Sul) potentiates SMO inhibitor, itraconazole (ITRA)-mediated inhibition of UVB-induced growth of BCCs in Ptch1+/−/SKH-1 mice Ptch1^+/−^/SKH-1 mice were treated with orally administered ITRA (40 mg/kg body weight; twice weekly) and topically applied Sul (80 mg/kg body weight; twice weekly) alone and in combination, 30 min prior to UVB irradiation (180 mJ/cm^2^) for 30 weeks. Data showing **A.** tumor volume/mouse (mm^3^). **B.** BCCs/mouse from ITRA- and Sul- treated, alone and in combination and vehicle-treated animals **C.** β-gal staining (magnification 20x), proliferation biomarker (PCNA) (magnification 40x) and TUNEL staining (magnification 40x) of BCCs in ITRA- and Sul-, alone and in combination- and vehicle-treated UVB-irradiated mice. **D.** Western blot analysis showing expression of PCNA, cyclin D1, Bax and Bcl2 in the skin of ITRA and Sul alone and in combination of UVB-irradiated Ptch^+/−^/SKH-1 mice. Boxes in histology of tumors show areas of high infiltration of hematopoietic cells which were further characterized in figure 6.

The inflammatory response in skin excised from chronically UVB-irradiated Ptch1^+/−^/SKH-1 mice showed that expression of 36 genes was enhanced, 24 genes down-regulated and the remaining 24 genes were unchanged (Figure 2B and Supplemental Figure S5B).

Treatment of chronically UVB-irradiated Ptch1^+/−^/SKH-1 mice with the classic SMO inhibitor cyclopamine (20 mg/kg body weight; twice a week), partially attenuated these UVB-induced gene expression patterns (Figure 2B and Supplemental Figure S5B). Only 17 of 84 genes in this panel reverted to baseline. Interestingly, Ccl3, Ccl4, Ccr3, Cxcl1, Pf4, Cxcl5, Il11, Il13, Il17b, Il1a and Il4 that had been altered by the introduction of Ptch1 heterozygosity were attenuated by cyclopamine treatment. However, the expressions of the majority of genes altered by UVB irradiation were substantially unresponsive to cyclopamine treatment. These data suggest that inflammatory signatures induced by activated Shh signaling and UVB are distinct and respond differentially to Shh pathway inhibitors. In this regard, Shh signaling has been implicated in the development of inflammatory responses in intestine and brain [42, 43]. Recently, Shh signaling has also been shown to be regulated by NFκB [44]. To our knowledge this is the first evidence that upregulation of Shh signaling contributes to a pro-inflammatory microenvironment in murine skin. It is of interest that Gorlin in an early publication had commented on the increased cutaneous prostaglandin E2 (PGE2) level in lesions from patients with NBCCS [45].

**Figure 6:**
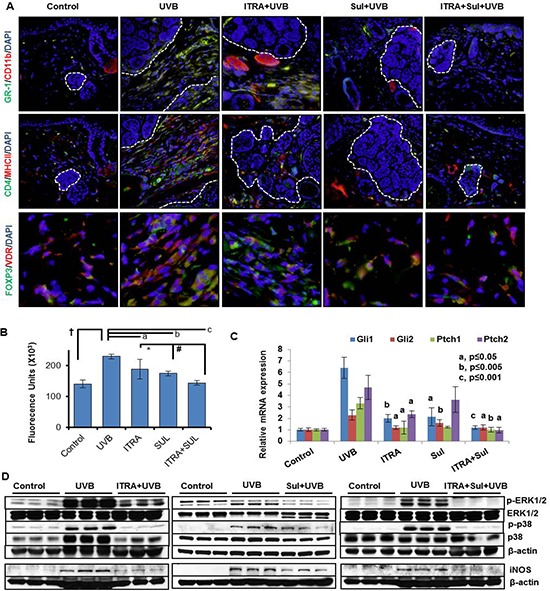
Combined administration of ITRA and Sul attenuates UVB-induced inflammatory responses in Ptch1+/−/SKH-1 mice **A.** immunofluorescence staining showing presence of GR-1 (green)/CD11b (red)-positive myeloid cells (upper panel), CD4 (green)/MHC11 (red)-positive T helper cells (middle panel) and FOXP3 (green)/VDR (red)-positive regulatory T cells (lower panel) in stroma of BCCs from UVB-irradiated age-matched control mice treated with ITRA and Sul alone and in combination. **B.** Cutaneous myeloperoxidase (MPO) activity in the skin from UVB-irradiated age-matched control mice and those treated with ITRA and Sul alone and in combination. † *p* = 0.0006; a *p* = 0.097; b *p* = 0.001; c *p* = 0.0002; *p = 0.118; #*p* = 0.02. **C.** Relative mRNA expression of Shh-signaling responsive genes. P value represents comparison of drug-treated vs UVB-irradiated mice. and **D.** Western blot analysis showing phosphorylation of ERK1/2 and p38 and expression of pro-inflammatory proteins iNOS in the skin from UVB-irradiated age-matched control, with ITRA and Sul alone and in combination treatment groups.

Next, we characterized the pro-inflammatory infiltrating hematopoietic cells in the BCC tumor microenvironment using immunofluorescence staining of various surface marker proteins that stain for these inflammatory cells. Compared to age-matched control skin, the underlying dermis of BCCs was rich in macrophages, neutrophils and bone marrow-derived GR-1^+^/CD11b^+^ myeloid cells, also known as myeloid-derived suppressor cells (MDSC). T helper cells (Th) and regulatory T cells (Treg) were also seen as shown in figure 6A, second panel. Exaggerated neutrophil infiltration in tumor-adjacent skin was also confirmed by markedly enhanced detection of neutrophil-derived myeloperoxidase (MPO) (Figure 6B). Prior studies have shown that MDSCs infiltrate the stroma of skin tumors [37, 46, 47]. In addition the presence of these cells in the tumor microenvironment of BCCs has recently been described by Fan et al who showed that CCL2/CCR2 are involved in the migration of these cells [48]. Our results also indicate an increase of CCR2 in tumor-adjacent skin. Similarly, in the skin of patients with NBCCS, microscopic basal cell lesions were found to be associated with infiltration of hematopoietic cells positive for CD11b/CD16 and CD11c/CD4 that characterize monocytes, macrophages, neutrophils, granulocytes, and natural killer cells as shown in figure 2E.

To further characterize the tumor microenvironment, we investigated the status of effector pro-inflammatory signaling pathways in the tumor-adjacent skin and BCCs in Ptch1^+/−^/SKH-1 mice. Earlier, we showed high expression of cyclooxygenase-2 (COX-2) in the stroma and tumor islands of human and murine BCCs [49]. Confirming these observations, enhanced COX-2 expression and inducible nitric oxide synthase (iNOS) also characterized these lesions. Additionally, consistent with previous observations showing association of MAP kinase activity with UVB-induced cutaneous inflammatory responses [50], here, we also found enhanced phosphorylation of the mitogen activated protein kinase (MAPK) signaling proteins Erk1/2 and p38 in tumor-associated tissue (Figure 6D).

Finally, we attempted to verify the role of eicosanoids in driving tissue inflammation in BCCs by assessing the effect of administering the non-steroidal anti-inflammatory drug (NSAID) sulindac (SUL). SUL treatment substantially reduced tumor-associated inflammation as confirmed by diminished infiltration of hematopoietic cells, reduced expression of iNOS, p-Erk1/2 and p-p38 in the tumor stroma (Figure 6D).

### Both spontaneous and UVB-induced BCCs in Ptch1^+/−^/SKH-1 mice carry point mutations in Ptch1 gene

To better understand the pathogenesis of the spontaneous BCCs in our Ptch1^+/−^/SKH-1 mice, we analyzed mutations in the tumor suppressor genes Ptch1 and p53. Humans with NBCCS inherit a germline mutation in one allele of the Ptch1 gene and tumor development is generally accompanied by loss of the remaining wild-type allele leading to aberrant activation of Shh signaling that drives the growth of these lesions [9, 11, 12, 16]. Here, we detected multiple Ptch1 mutations in spontaneous BCCs from Ptch1^+/−^/SKH-1 hairless mice (Supplemental Figure S6) which were similar to those known to occur in NBCCS patients [51]. However, we could not detect any mutations in the p53 DNA binding region of these spontaneous BCCs. In contrast, UVB-induced BCCs did manifest UVB-signature p53 mutations in addition to mutations in Ptch1 (data not shown).

### Ptch1^+/−^/SKH-1 mice are highly sensitive to IR

Patients with NBCCS are known to be exquisitely sensitive to IR. In the past, NBCCS patients with childhood medulloblastomas were treated with IR and later in life often developed large numbers of BCCs in irradiated skin sites [52]. To demonstrate the close resemblance of Ptch1^+/−^/SKH-1 hairless mice with NBCCS patients, we irradiated these animals with a single dose of IR (5 Gy). Similar to patients with NBCCS, these animals developed multiple BCCs over the dorsal and ventral skin surface starting from week 10 (Figure 3A). By week 19 virtually all of the animals had developed large numbers of tumors, (approximately 70–80 tumors/mouse) (Figure 3B) accounting for a total tumor volume exceeding 3500 mm^3^ (Figure 3C). Histological examination showed sheets of microscopic BCCs spread throughout the dermis. (Figure 3D and Supplemental Figure S7A and S7B). The majority of these BCCs exceeded 4–5 mm in diameter showing increased expression of proliferation markers including cyclin D1 and PCNA along with elevated anti-apoptotic Bcl2 (Figure 3E). There was also elevated mRNA expression of Shh-signaling pathway genes Gli1, Gli2, Gli3 and Ptch1/2 in both tumor-adjacent skin and BCCs of IR-irradiated mice (Figure 3F). In general, increased expression of cyclins D1, D2, D3 and E was detectable in these tumors at the protein and mRNA levels (Figure 3F). By contrast, Ptch1^+/−^ mice in C57BL/6 or mixed genetic backgrounds when exposed to an identical single dose of IR developed no more than 1 to 3 BCCs/mouse during their lifetime [24].

**Figure 7:**
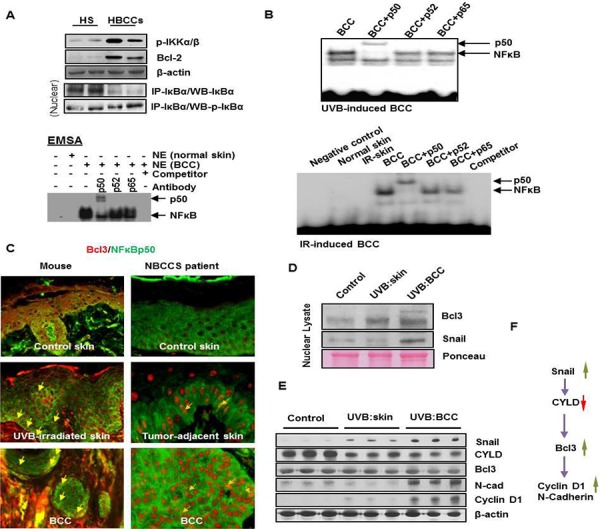
Activation of non-canonical NFκB signaling pathway in human and murine BCCs **A.** Activation NFκB in human BCCs as shown by co-immunoprecipitation, EMSA and super-shift assays. **B.** EMSA/super-shift assays showing the activation of NFκB in UVB- and IR-induced murine BCCs. **C.** Co-expression of Bcl3 and p50 in mouse BCCs and in BCCs obtained from patients with NBCCS. Co-nuclear localization of Bcl3 and p50 proteins representing yellow nuclear staining (original magnification 40X). **D.** Western blot analysis showing expression of Bcl3 and Snail in nuclear lysates prepared from BCCs and tumor-adjacent skin; Expression of Bcl3 downstream transcription targets of Cyclin D1 and N-cadherin. **E.** Western blot showing expression of Snail, Cylindromatous (CYLD), Bcl3, N-cadherin and Cyclin D1 in whole cell lysates prepared from UVB-induced BCCs and tumor adjacent skin. **F.** Flow diagram showing regulation of Bcl3 and its downstream targets Cyclin D1 and N-cadherin.

### Smo inhibitors incompletely inhibit BCC growth in chronically UVB-irradiated Ptch1^+/−^/SKH-1 mice

Cyclopamine is a naturally occurring Smo antagonist [53, 54]. In prior studies, we initially showed that chronic oral administration of cyclopamine reduces UVB-induced BCCs in Ptch1^+/−^/C57BL/6 mice or in Ptch1^+/−^ mice of mixed genetic background [54]. Thereafter, Genentech developed an analog of cyclopamine known as vismodegib (GDC-0449 or Erivedge). This drug is a more potent Smo inhibitor and binds to Smo at a site identical to that of cyclopamine [32, 33, 55, 56]. To test the potency of vismodegib in blocking growth of BCCs in our murine model of NBCCS, we administered vismodegib (40 mg/kg body weight by mouth) to Ptch1^+/−^/SKH-1 mice thirty minutes prior to each exposure to UVB and assessed its protective effects against UVB-induced BCCs. As shown in Figure 4, vismodegib decreased both macroscopic and microscopic BCCs in these animals. Vismodegib attenuated development of UVB-induced macroscopic BCCs by 73% (*p* = 0.001) and tumor volume by 76% (*p* = 0.004) (Figure 4A and 4B). Consistently, microscopic BCCs were reduced by at least 60–70% (*p* = 0.037) (Figure 4C). Reductions in tumor burden were accompanied by diminished PCNA expression and enhanced numbers of TUNEL-positive apoptotic cells (Figure 4D). Interestingly, tumor stromal cells were also frequently TUNEL-positive (Figure 4D, lower right panel). These data demonstrate that vismodegib is a potent but incomplete inhibitor of the growth of BCCs in Ptch1^+/−^/SKH-1 mice. We next compared the inhibitory effect on Shh-signaling and the anti-tumor efficacy of vismodegib cyclopamine and ITRA, by analyzing samples from various treatment groups with qPCR for the expression of Shh signaling genes as shown in figure 4E. Chronic treatment with these agents for 30 weeks diminished Shh signaling by an average of 80, 60 and 47%, respectively by vismodegib, ITRA and cyclopamine However, differences in their ability to inhibit BCC growth (both microscopic and macroscopic) were subtle (60–70%) and did not correlate with their inhibitory effect on Shh pathway genes (figure 4E).

### Combined administration of inhibitors of Shh signaling and NSAIDs substantially abrogates the growth of UVB-induced BCCs in Ptch1^+/−^/SKH-1 mice

Our group has recently participated in a placebo-controlled randomized clinical trial in patients with NBCCS showing that orally administered vismodegib substantially reduces BCC burden in slightly more than half (56%) of these patients. Unfortunately these anti-tumor effects were largely reversible following discontinuation of drug administration [57, 58]. Our results show that vismodegib partially eliminates UVB-induced BCCs in our Ptch1^+/−^/SKH-1 mice (Figure 4). SUL was also only partially effective in this regard confirming our earlier observations with the specific COX-2 inhibitor, celecoxib which showed partial efficacy both in a murine model of sporadic BCCs and in patients with NBCCS [59]. Recently, ITRA, an FDA-approved azole antifungal drug, was shown to be a potent SMO inhibitor with an IC_50_ of approximately 800 nM in the Shh-Light2 reporter cell line [34]. ITRA is an oral antifungal agent commonly used in clinical practice that is administered over a period of months [34]. ITRA binds to Smo at a site distinct from that of cyclopamine or cyclopamine analogs [60] and also manifests potent activity against the vismodegib resistant SMO mutant, human SMO^D473H^/murine SMO^D477G^ [32, 61]. In this study, we assessed the ability of combining orally administered ITRA (40 mg/kg body weight) and SUL (80 mg/kg body weight) on the growth of BCCs in our Ptch1^+/−^/SKH-1 mice. To further facilitate the data interpretation, we have utilized various control groups including age-matched non-drug non-irradiated, UVB-irradiated alone, ITRA treated alone+UVB irradiated and SUL-treated alone+UVB-irradiated. In these experiments, the combination of ITRA and SUL almost completely eliminated UVB-induced growth of BCCs. Tumor volumes were reduced by 50% (ITRA alone), 63% (SUL alone) and 90% (ITRA and SUL combined (Figure 5A). The combined regimen diminished UVB-induced BCCs by 92% from 1.8 ± 0.38 to 0.13 ± 0.09 BCCs/mouse as compared to single treatment (ITRA, 0.8 ± 0.22 BCCs/mouse; SUL, 0.9 ± 0.31 BCCs/mouse) (Figure 5B). Histological and immunohistological analysis of these tumors confirmed the superior efficacy of the combined ITRA and SUL regimen and showed reduced staining for proliferation markers and increased numbers of TUNEL-positive cells (data not shown). Microscopic BCCs were reduced by 67% (ITRA alone), 58% (SUL alone and 72% (combined ITRA and SUL) (Figure 5C, upper panel). Similar results were seen with microscopic BCCs as well (Figure 5C, middle and lower panel). ITRA alone and SUL alone decreased expression of biomarkers of cell proliferation (PCNA and cyclin D1), and increased expression of pro-apoptotic biomarkers (Bax) in BCCs and the combined ITRA and SUL regimen was again more effective in this regard (Figure 5D).

We also used a similar approach to assess the tumor-associated inflammatory response during tumor development. Compared to treatment with ITRA and SUL alone, the combination was again more effective in reducing the inflammatory response. As shown in Figure 6A and Supplemental Figure S8A and S8B, GR-1/CD11b-myeloid cells, CD4, MHCII, FOXP3 and vitamin D receptor (VDR)-positive Treg cells were diminished in the tumor microenvironment surrounding microscopic BCCs. The number of infiltrating neutrophils as reflected by MPO enzyme activity was also significantly (*p* < 0.001) reduced in animals receiving the combination (Figure 6B). These changes in the inflammatory cell infiltrate were accompanied by a striking reduction in the phosphorylation-dependent activation of Erk1/2 and p38 MAPKs, and diminished iNOS expression (Figure 6D and Supplemental Figure S9). These signaling proteins are known to be expressed in cutaneous inflammation including that induced by UVB as described earlier [50]. SUL-mediated reduction in inflammatory response was also accompanied by significant decrease in Gli1. Gli2 and Ptch1 but not Ptch2 showed some decrease whereas combination of ITRA+SUL was much more effective in this regard (Figure 6C). Based on the enhanced anti-tumor efficacy of the combined regimen in our mouse model we anticipate conducting a clinical trial using this approach in our cohort of NBCCS patients.

**Figure 8:**
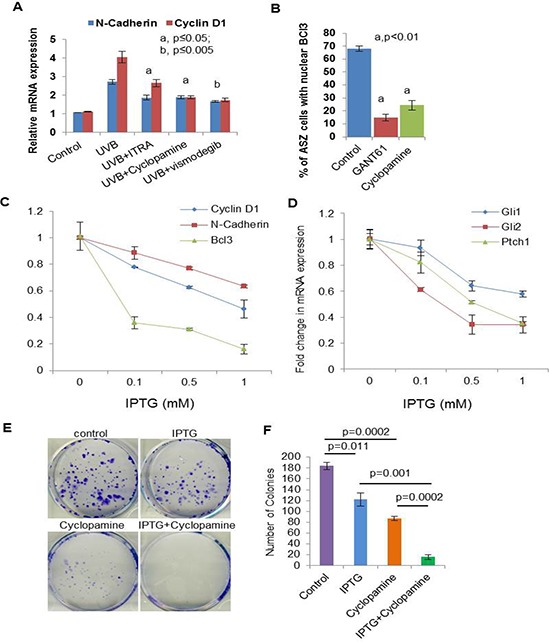
Crosstalk of Shh and Bcl3 signaling pathways in BCC cells regulate proliferation **A.** mRNA expression of N-cadherin and cyclin D1 in skin of UVB-irradiated and Shh-inhibitors-treated. **B.** Percentage of ASZ001 cells showing nuclear localization of Bcl3 in GANT61 (10 μm) and Cyclopamine (5 μm)-treated cells. **C.** TaqMan real-time PCR showing effects of Isopropyl β-D-1-thiogalactopyranoside (IPTG) treatment on the expression of Bcl3, CyclinD1 and N-cadherin in ASZ001 cells. ASZ001 cells have been infected with IPTG-inducible Bcl3 shRNA lentiviral vector and were selected following treatment with puromycin containing growth medium. **D.** TaqMan real-time PCR showing the expression of shh signaling related genes Gli1, Gli2 and Ptch1 following knockdown of Bcl3 in ASZ001 cells. **E.** Colony formation assay in control, IPTG (1 mM), Cyclopamine (5 μm) and IPTG+Cyclopamine treated ASZ001 cells. **F.** Bar diagram showing colony numbers following various treatments.

**Figure 9:**
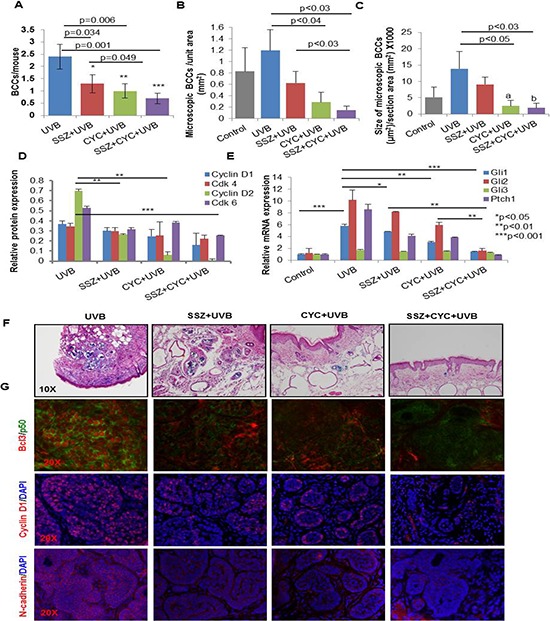
Blocking NFκB and Shh signaling respectively with sulfasalazine (SSZ) and cyclopamine (CYC) reduces growth of BCCs in Ptch1^+/−^/SKH-1 mice **A.** Effects of SSZ and CYC alone and in combination on UVB-induced BCCs. Effects of these treatments on the number (B) and size of microscopic BCCs (C). **D.** Relative expression of cyclin D1/2 and their partner kinases CDK4/6 in various treatment groups. **E.** mRNA expression of Shh signaling-related genes in various treatment groups as described in **A. F.** Representative photograph showing microscopic BCCs in various treatment groups (blue staining) represents β-gal staining); **G.** representative images of Bcl3 / N-cadherin / Cyclin D1 immunofluorescence staining in various treatments.

### NFκB signaling pathway influences the pathogenesis of inflammatory responses associated with the growth of BCCs

To define the mechanism whereby cutaneous inflammation accelerates the growth of BCCs, we tested whether crosstalk between the NFκB and Shh signaling pathways might be involved. NFκB-dependent signaling is known to mediate inflammatory responses during progression of neoplasia [62]. Crosstalk between these two pathways has been postulated to contribute to the pathogenesis of various epithelial tumors [63–67]. As an initial approach, we assessed NFκB transcriptional activity in human and murine BCCs. Using electrophoretic mobility shift assay (EMSA), we found that NFκB was consistently up-regulated in both murine and human BCCs (Figure 7A). However, supershift assays detected only the p50 subunit of NFκB in these tumors. These effects were consistently seen in spontaneous BCCs and in IR or UVB induced BCCs (Figure 7B). In human BCCs, the upstream kinase, IKKα/β, and downstream target genes, Bcl2, TRAIL and IL1β were all upregulated. Inhibitory kappa B α (IκBα) was decreased whereas its phosphorylated form was increased (figure 7A). In canonical NFκB pathway, phosphorylation of IκBα is required for its dissociation from the cytosolic transcriptionally inactive heterotrimeric NFkB complex, which then migrates to the nucleus as a transcriptionally active heterodimeric protein complex as shown in figure 11.

It is known that the p50 subunit of NFκB lacks a DNA transactivating domain [68]. However in considering the role of a non-canonical pathway, it could acquire transcriptional activity by engaging another partner protein known as Bcl3, an atypical member of the IκB family [69]. Bcl3 is known to associate with nuclear p50 or p52 homodimers and modulate transcription [69]. We utilized immunofluorescence techniques to determine whether p50/Bcl3 and p52/Bcl3 complexes are present in murine and human BCCs. Co-localization signals (Figure 7C and data not shown) were detected for p50/Bcl3 nuclear complexes in BCCs obtained from patients with NBCCS as well as in our mouse model. However, in a small subset of human BCC cells, nuclear staining for p65 was also positive (data not shown). This confirms the results of western blot analysis of nuclear extracts prepared from these human BCCs, which also showed a signal for p65 (Supplemental Figure S10A). These data support the concept that in murine BCCs, Bcl3-protein complexes primarily regulate transcriptional activity whereas in human BCCs both Bcl3 and p65 complexes with p50 or p52 regulate transcriptional activity.

Bcl3 expression is influenced by multiple factors [69–71]. These include cylindromatous (cyld) protein and/or the Th2 tumor microenvironment as shown by the Flow Diagram in Figure 7F. We found that cyld which regulates the status of ubiquitinated Bcl3 was downregulated in BCCs (Figure 7E). We also observed that similar to human BCCs [72], the tumor stroma of murine BCCs show characteristic features of a Th2 microenvironment including increased expression of IL4 and the presence of cells expressing CD4/FOXP3 (Figure 6A and Supplemental Figure S8A & S8B and S10B). Th2 cytokines are known to up-regulate Bcl3 expression in murine skin [73].

To demonstrate that crosstalk between p50-Bcl3 and Shh signaling underlies the pathogenesis of BCCs in NBCCS, we employed multiple approaches as shown in figure 8. First, we treated murine BCC ASZ cells with Shh inhibitors cyclopamine and GANT61 as shown in figure 8A. While cyclopamine inhibits Shh signaling by binding with Smo, GANT61 inhibits its downstream target transcription factors Gli1 and Gli2 [54, 74]. Treatment with both of these agents significantly diminished nuclear Bcl3-positive cells. Next, we assessed Bcl3 expression in tumors obtained from vismodegib, ITRA and cyclopamine-treated animals. Decreased expression of p50-Bcl3 and -dependent transcription-associated targets such as cyclin D1 and N-cadherin were observed (Figure 8A). Finally, we investigated whether shRNA-based Bcl3 knock-down diminishes Shh signaling (Figure 8B, 8C and 8D and Supplemental Figure S11). As shown in figure 8E and 8F, the isopropyl β-D-1-thiogalactopyranoside (IPTG)-mediated shRNA-targeting of Bcl3 in ASZ001 cells reduced colony numbers and sizes in a colony forming assay. Combining Bcl3 knock-down with cyclopamine treatment, colony size and number were reduced to <5% of scrambled RNA transfected cells. Similarly, we observed substantially reduced expression of Gli1/2 and Ptch1 in Bcl3 knock-down BCC ASZ001 cells (Figure 8C) confirming the existence of crosstalk between these two pathways.

As we could not identify a small molecule that could specifically block p50/Bcl3 signaling, we employed sulfasalazine (SSZ), an inhibitor of upstream IKKα/β [75]. SSZ is also an FDA-approved drug for the treatment of psoriatic arthritis [76, 77]. Treatment with SSZ alone and in combination with cyclopamine diminished the growth of UVB-induced BCCs in Ptch1^+/−^/SKH-1 mice. Both BCC numbers and volumes were significantly reduced (Figures 9A, 9B and 9C). This reduction in tumorigenesis was associated with the inhibition in p-IKKα/β expression (Supplemental Figure S13A) and diminished cell cycle regulatory cyclinD1/2 and the partner kinases CDK4/6 and reduced proliferative signaling as assessed by PCNA expression (Figure 9D and Supplemental Figure S12). In this case also, we observed that combining SSZ+cyclopamine was much more effective (*p* < 0.001) than either alone. Interestingly, we further confirmed that Shh and NFκB pathways manifest crosstalk as SSZ partially reduced Shh signaling (Figure 9E) whereas cyclopamine treatment reduced nuclear Bcl3 and dependent signaling (Figure 9G and Supplemental Figure S13B), while a combination of the two almost completely abrogated the activation of both of these pathways (Figure 9E and 9G, second panel).

**Figure 10:**
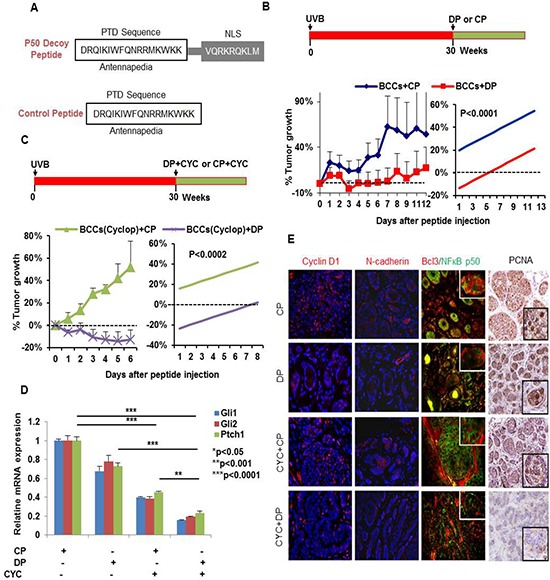
Blocking NFκB with p50 decoy peptide (DP) reduces growth of BCCs in Ptch+/−/SKH-1 mice **A.** Sequence of p50 DP and CP. **B.** Tumors treated with CP were unaffected and continue to grow whereas DP treatment was tumor growth static. **C.** Animals treated with the combination of cyclopamine and DP showed regression of tumors as compared to mice treated with cyclopamine+CP (*p* < 0.0002). **D.** Relative mRNA expression of Gli1/2 and Ptch1 in tumor lesions treated with CP, DP and/or Cyclopamine. **E.** Nuclear localization of Bcl3/p50 and for the levels of Cyclin D1/N-cadherin/PCNA (immuno-fluorescence and immunohistochemical staining). N-cadherin and cyclin D1 are known downstream transcriptional targets of Bcl3/p50. Treatment with DP blocked nuclear entry of Bcl3 (red)/p50 (green) and reduced Cyclin D1 and N-cadherin expression whereas treatment with CP failed to do so. Please note nuclear signal (yellow) for the co-localization of Bcl3/p50 in CP-treated control panel whereas DP-treated tumors show nuclear staining for these proteins. Simple linear regression for the analysis of the differences between the two treatment groups was used. The outcome is the averaged value of the tumor decrease percentage compared with day 0 for different tumors. We considered the treatment effect and the time variable as the covariates in our regression model and considered statistically significant with *p*-value = 0.0002 or less.

To further strengthen the role of the p50/Bcl3 transcriptional pathway in the pathogenesis of BCCs, we injected a p50 decoy peptide (DP) or a control peptide (CP) directly into macroscopic BCCs once daily for two weeks. The DP contained a protein transduction domain (PTD) antennapedia sequence that allowed it to penetrate the plasma membrane as well as a nuclear localization sequence (NLS) [78]. The CP contained only the PTD antennapedia sequence (Figure 10A). We observed that DP-injected tumors showed enhanced growth arrest as compared to tumors injected with CP (Figure 10B). To determine whether SMO inhibition combined with NFκB inhibition is more effective in inhibiting tumor growth, we performed an identical experiment in mice treated with cyclopamine (Figure 10C). Indeed cyclopamine-treated-animals were more responsive to the growth inhibitory effects of DP (Figure 10C), and manifested enhanced tumor regression. Consistently, in both experiments, we found that DP treatment blocked the nuclear localization of Bcl3 and p50/Bcl3-dependent transcriptional activity. Thus, the downstream targets of Bcl3, including cyclin D1 and N-cadherin were substantially decreased in the shrinking lesions (Figure 8E). Again, here also we confirmed the crosstalk of Bcl3/Shh signaling pathways. DP-treatment alone diminished Shh signaling (figure 10D) and cyclopamine reduced nuclear Bcl3 staining (figure 10E). These results suggest the existence of crosstalk between the two pathways and also demonstrate that p50/Bcl3-dependent transcriptional activity supports BCC tumor growth in this murine model and combined blockade of NFκB and Shh signaling is substantially more effective in abrogating the growth of BCCs in a mouse model of NBCCS.

**Figure 11:**
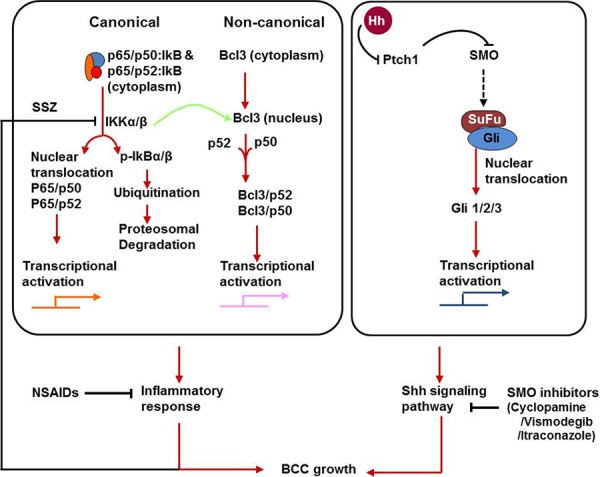
Flow diagram showing canonical and non-canonical NFκB pathways Nuclear localization of p50 and Bcl3 complex and its transcriptional activation drives overexpression of downstream target genes such as Cyclin D1 and N-cadherin. Moreover, activation of both Shh and p50/Bcl3 signaling pathways in basal keratinocytes drive pathogenesis of BCCs in the murine model of NBCCS and in patients with NBCCS. Inhibition of these two pathways together abrogates BCC development in Ptch1^+/−^/SKH-1 mice.

## DISCUSSION

NBCCS is an autosomal dominant disorder with an estimated incidence of 1:57,000 [36, 79]. While sporadic NMSCs including SCCs and BCCs are common in Caucasian populations, the pathogenesis of BCCs in patients with NBCCS is known to occur in multiple ethnic groups of varying degrees of skin pigmentation *albeit* with varying penetrance [14, 79]. However, Caucasians typically develop higher numbers of BCCs as compared to other darker races [13, 80]. Thus, genetic background may influence disease penetrance as demonstrated by predisposition and severity of symptoms, including developmental defects and the growth of skin and extracutaneous neoplasms. A relevant animal model that encompasses the phenotype of NBCCS has been slow to develop and currently no murine model exists that closely manifests the exact pathophysiology of the disease [24, 27, 52]. To date, the available murine models manifest only selected features of NBCCS but more closely resemble sporadic BCCs [24, 27]. Furthermore, penetrance of the disease in these models is only 10–15% [24, 27]. Considering the known dependence of severity and propensity of disease symptoms on genetic background in humans, we developed a murine model that closely resembles NBCCS by transferring Ptch1 heterozygosity from a tumor-resistant mouse strain (C57BL/6) to SKH-1, a strain that is highly susceptible to skin tumorigenesis [26]. As is the case with NBCCS patients who typically develop multiple BCCs on both sun-exposed and sun-protected body areas, our Ptch1^+/−^/SKH-1 mice develop spontaneous BCCs with high frequency (incidence of 40%) in any anatomic area over their lifetime. Similar to NBCCS patients these animals develop BCCs early in life and they also are susceptible to other Shh-driven internal malignancies including RMS and to a lesser extent medulloblastomas [15, 81]. We found that a single exposure of Ptch1^+/−^/SKH-1 mice to IR (5 Gy) results in hundreds of BCCs over their entire body including both dorsal and ventral skin, thereby recapitulating the fact that exposure of NBCCS patients to therapeutic IR has led to field cancerization [24, 82]. Histological examination of the skin of the IR-treated Ptch1^+/−^/SKH-1 mice revealed numerous BCCs in a pattern identical to that occurring in the IR-treated NBCCS patients [36, 79].

The major underlying molecular defects involved in the pathogenesis of NBCCS and in experimental animals are Ptch1 gene mutations. Besides being the receptor for hh ligands, Ptch1 is a potent tumor suppressor in the skin [9, 20, 54]. Mutant Ptch1 with diminished or absence of tumor suppressor activity results in the growth of BCCs in humans as well as in experimental animal models [9, 20, 54]. These Ptch1 mutations also occur in patients with sporadic BCCs. However, in patients with NBCCS, germline allelic loss coupled with multiple mutations in the remaining wild-type Ptch1 allele typically leads to the appearance of large numbers of BCCs and other extracutaneous malignancies [11, 36, 79]. Molecular characterization of BCCs in Ptch1^+/−^/SKH-1 mice which carry only one mutant allele of Ptch1 show augmented expression of Shh signaling-related genes and enhanced expression of cell cycle regulatory proteins essential for accelerated cell proliferation. In addition, consistent with the known molecular pathogenesis of UVB-induced skin cancer, both Ptch1 and p53 mutations (C-T transversion) are common in BCCs induced in chronically UVB-irradiated Ptch1^+/−^/SKH-1 mice. Cyclobutane pyrimidine dimers are the major DNA photoproduct formed by UVB including cytosine to thymine (C to T) transitions when two pyrimidines are adjacent to each other or, at times, CC to TT transitions and both types lead to mutations designated as signature mutations for UV damage [4, 5].

Cyclopamine is an alkaloid that was isolated from the American Corn Lilly, a plant that grows wild in the Northwestern United States. It was known to be a classical Smo inhibitor and has been shown to block UVB-induced Shh signaling and BCC development [53, 54]. The FDA-approved drug vismodegib, an analog of cyclopamine that was developed by Genentech, is a more potent SMO inhibitor as compared to cyclopamine despite both binding to Smo at the same site [33, 55, 83]. The FDA-approved triazole antifungal drug ITRA is also a SMO inhibitor that binds the molecule at a site distinct from that of cyclopamine or vismodegib [34, 60]. The emergence of ITRA as a clinically useful SMO inhibitor is of particular interest and importance. Because this antifungal drug has a long record of clinical safety, it has excellent potential for use as a systemic agent for the chemoprevention of BCCs in NBCCS [34, 35]. A major limitation of vismodegib is the frequent development of drug resistance as a result of acquired Smo mutations that reduce its SMO- binding affinity [32, 57, 84]. Since ITRA binds to SMO at a different site, its efficacy seems to be unaffected by these vismodegib-associated mutations [60]. In this study, we utilized our Ptch1^+/−^/SKH-1 mice to assess the efficacy and safety of both vismodegib and itraconazole. Each of these was only partially effective in inhibiting the growth of BCCs and these results closely mimic those observed in clinical trials of vismodegib and ITRA in patients with NBCCS [33, 35, 57].

In one of his classical reviews of NBCCS published in 1987 Gorlin pointed out that “…independent observations of increased prostaglandin levels associated … with aggressiveness of basal cell cancers merit further investigation both as a fundamental cellular mechanism and as a possible basis for treatment (e.g., with antiprostaglandins)”. The cyclooxygenases (COXs) are arachidonic acid-metabolizing enzymes that generate prostaglandins involved in the pathogenesis of inflammatory responses including those induced in the skin following UVB exposure [85–87]. In particular UVB irradiation of the skin is known to induce COX-2 thereby, enhancing the prostaglandin E2 production that drives cell proliferation and neoplastic growth [88]. Not surprisingly, NSAIDs have shown some efficacy as cancer chemopreventive agents [85, 88–90]. We previously showed that COX-2 is highly expressed in the stroma of both human and murine BCCs emphasizing its likely contribution to tumor growth perhaps by altering the tumor microenvironment [59]. Importantly, Shh signaling also modulates the tumor stroma and tumor-associated microenvironment [91–94]. Furthermore, employing both genetic and pharmacological approaches, we showed in a murine model of UVB-induced skin carcinogenesis and in patients with NBCCS that overexpression of COX-2 accompanies accelerated BCC growth whereas NSAID-induced COX-2 inhibition by celecoxib partially (30–50%) abrogates growth of these tumors [59, 85, 95].

Based on the known risk of cardiac events in patients treated with ‘Coxibs’ [96] and also reported resistance to these drugs in a small subset of the human population [97], in this study we have also assessed the chemopreventive effects of sulindac, a known COX inhibitor in our Ptch1^+/−^/SKH-1 mice and show that it has partial efficacy in reducing the growth of UVB-induced BCCs. Inhibiting inflammation alone or Shh signaling alone is only partially effective in blocking the growth of BCCs in our murine model or in humans with NBCCS. These results further emphasize that Ptch1^+/−^/SKH-1 mice not only recapitulate the global phenotypic pattern and the molecular pathogenesis of BCCs in NBCCS but also mimic the responses to chemopreventive and chemotherapeutic agents similar to those observed in patients with this syndrome. In further studies we have clearly shown that by combining a Shh inhibitor (vismodegib or ITRA) and a COX inhibitor (the NSAID Sulindac) it is possible to obtain superior anti-tumor efficacy as compared to either class alone in treating BCCs in our murine model of NBCCS (Figure 11). Our results indicate that this combination almost completely blocks the growth of these lesions, suggesting that combinatorial regimens utilizing Smo inhibitors and NSAIDS offer potentially superior benefit for patients with NBCCS. It is important to emphasize that sulindac is being employed as one of the chemopreventive drugs in a recently NCI-funded “Adenoma and Second Primary Prevention Trial (ID # S0820).

In the course of these studies we also searched for the possible involvement of pro-inflammatory signaling pathways in the pathogenesis of BCCs. NFκB is a transcription factor that has been closely linked to the pathogenesis of cutaneous inflammation and neoplasia including that induced by UVB [71, 72]. Crosstalk between NFκB and Shh signaling has been described in a variety of experimental settings [63–65, 92]. UVB has been shown to activate NFκB transcriptional activity during skin tumorigenesis [98]. Since, we have observed augmented basal and UVB-induced inflammatory responses in Ptch1^+/−^/SKH-1 as compared to the parental Ptch1^+/+^/SKH-1 strain, we postulated that NFκB activation may contribute to the pathogenesis of BCCs. Cytosolic inactive NFκB is a trimeric protein complex consisting of p65/p50/IkB and/or p65/p52/IkB complexes as described in figure 11. Following various stimuli, phosphorylation-dependent degradation of inhibitory IkB occurs, leading to the release of transcriptionally active p65/p50 or p65/p52 protein complexes, that migrate to the nucleus where they can bind to promoters of genes that drive expression of pro-inflammatory signaling pathways [99, 100]. Indeed, we found enhanced expression of phosphorylated IkB during the pathogenesis of humans BCCs and BCCs that develop in our mouse model.

NFκB is known to transcribe a diverse array of genes including those that orchestrate tissue inflammation, cell proliferation and inhibition of apoptotic responses during cancer pathogenesis [41, 62, 99]. Unlike, the known involvement of canonical p65-dependent NFκB transcriptional activity in the growth of UVB-induced SCCs [98, 101], we show here enhancement of Bcl3-regulated non-canonical NFκB signaling in BCCs; furthermore we also found similar nuclear localization of the p50-Bcl3 complex in BCCs from patients with NBCCS. These studies reveal the importance of Bcl3 in the pathogenesis of this neoplasm. Bcl3 is known to be involved in diverse physiological and pathophysiological processes {For review please see [102]}. It serves as an oncogene in the pathogenesis of certain neoplasms [103–106]. It also regulates TLR-mediated macrophage responses [71]. We discovered here an additional novel aspect of this signaling which indicates the involvement of crosstalk with Shh pathway in promoting the pathogenesis of BCCs.

The exact mechanism that regulates Bcl3 expression is not clear [107]. However, multiple pathways are known to modulate Bcl3 expression [69, 107] including Th2 cytokines [73]. Furthermore human BCCs manifest a Th2 dominant tumor microenvironment [72]. We observed a similar Th2 microenvironment in UVB-induced murine BCCs in our Ptch1^+/−^/SKH-1 mice. Bcl3 expression is also known to be modulated by cyld protein, a deubiquitinating enzyme [108]. Mutated cyld drives the pathogenesis of cylindromas, an unusual benign human skin neoplasm of hair follicle origin [109]. Cyld inhibits tumor cell proliferation by blocking Bcl3-dependent NFkB signaling. Reduced expression of cyld was previously reported in BCCs [109, 110] and was also confirmed in this study.

Since we could not identify a specific inhibitor of the Bcl3-p50 pathway, we attempted an alternative approach. The upstream kinase IKKα/β which often regulates NFκB signaling and modulates the pathogenesis of cancer development, has been identified as an important molecular target for the cancer treatment and prevention [75]. It was also reported that SSZ, an FDA-approved drug used for treating psoriatic arthritis [76, 77], is a potent inhibitor of this kinase [111, 112]. In this study, we employed this drug to clarify the role of Bcl3 in the pathogenesis of BCCs in our murine model. We observed that SSZ reduces nuclear accumulation of Bcl3 and inhibits the growth of BCCs. Furthermore combining SSZ with cyclopamine enhanced the anti-tumor efficacy.

Bcl3's involvement in the pathogenesis of BCCs was further confirmed by our studies showing that intra-tumoral injections of a cell permeable p50 decoy peptide (DP) blocked nuclear translocation of Bcl3 and retarded BCC growth. Use of this DP specifically blocks nuclear translocation of Bcl3 [78] thereby reducing its ability to transcriptionally activate BCC cell proliferation. Despite concerns about the specificity of this approach [113], we believe that the multiple studies performed here provide a strong support to the concept that both Shh and Bcl3-dependent non-canonical NFκB may be crucial for the pathogenesis of BCCs particularly in patients with NBCCS. Reduced expression of cyclin D1/N-cadherin in residual tumors coupled with static tumor growth confirmed the importance of p50/Bcl3 in the growth of BCCs in our Ptch1^+/−^/SKH-1 mice. Moreover, tumor regression by the combined inhibition of Shh and Bcl3 signaling pathways suggests that targeting these two pathways might substantially enhance anti-tumor efficacy in our NBCCS model system (For summary please see figure 11). p50 DP can also block p65 transcriptional activity in tumor infiltrating macrophages [114] suggesting that this pathway may also be involved in inhibiting tumor growth. Increased Bcl3 expression occurs in a variety of cancers [105, 107, 115] and some non-cancer-related inflammatory skin conditions such as contact dermatitis [73, 116]. Our observations that Bcl3 knockdown inhibits Shh signaling and blocking Shh signaling with multiple agents diminishes Bcl3-dependent signaling point toward meaningful crosstalk between the Shh and Bcl3-dependent signaling pathways in the pathogenesis of BCCs in this murine model and potentially in NBCCS patients.

In summary, our studies describe a novel and unique murine model for NBCCS that will permit more detailed investigation of the molecular pathogenesis and chemoprevention of BCCs in patients with this dominantly inherited disorder. The studies described here provide a compelling rationale for prospective clinical trials using combinations of small molecular weight inhibitors of Smo and COX in patients with NBCCS to reduce tumor burden. These studies also highlight the importance of a *hitherto* undescribed Bcl3-signaling in the pathogenesis of BCCs that are relevant to our experimental model of NBCCS and to patients with this disease.

## MATERIALS AND METHODS

### Ptch1^+/−^/SKH-1 hairless mice

Male breeders (6–7 weeks old) of Ptch1^+/−^/C57BL/6 mice which were developed by deletion of exons 1 and 2 and insertion of the LacZ gene at the deletion site as described previously were purchased from Jackson Laboratory (Bar Harbor, ME) [26, 27]. The female breeders of SKH-1 hairless strain (5–6 weeks old) were purchased from Charles River Laboratories, Massachusetts and acclimatized for a week as described earlier [86]. One male and two female mice were placed together in individual cages for mating. After confirming pregnancies, the males and females were separated. Twenty-one days after birth, the litters were separated from their mothers. At the age of 4 weeks, these animals were sexed and separated. The litters were genotyped by tail clipping at the age of 10–11 days using primers TGGCTGAGAGCGAAGTTTCAG and TTCCACCCACAGCTCCTCCAC for wild-type and GGATGATCTGGACGAAGAGC and AGAAGGCGA TAGAAGGCGAT for truncated alleles. Ptch1^+/−^/SKH-1 hairless mice (F2) were selected for backcrossing onto SKH-1 hairless mice again to minimize the C57BL/6 genetic background for an additional 9 generations, resulting in a colony of Ptch1^+/−^/SKH-1 mice as described in Supplemental Figure 1. Data presented from various experiments in this study were generated employing mice between 10 and 25 generations.

### UV light source

A UV Irradiation Unit (Daavlin Co., Bryan, OH) was employed for these studies. The UVB source consisted of 6 Broadband UVB lamps ranging from 290 to 320 nanometers. We employed a Kodacel cellulose film (Kodacel TA401/407) to eliminate UVC radiation. A UVC sensor (Oriel's Goldilux UVC Probe, Stratford CT) was used routinely during each exposure to confirm lack of UVC emission. The dose of UVB was quantified with X-96 Meter from Daavlin. The radiation was further calibrated with an ILI700 Research radiometer/photometer from International Light Inc. (Newburyport, MA). The distance between the radiation source and targets was maintained at 25 cm. The irradiation assembly was kept in an air-conditioned room with an additional fan to maintain the ambient temperature.

### UVB-irradiation protocol and treatments

Ptch1^+/−^/SKH-1 hairless mice (10–25 generation) were used for UVB-irradiation and various treatments (unless described otherwise) which were divided into different groups of 20 mice each. Mice left untreated, served as age-matched control (negative control). Mice were irradiated with UVB (180 mJ/cm^2^; twice/week) and treated with vehicle control, served as positive control. Whereas, test groups were administered with cyclopamine (20 mg/kg body weight; I.P.) or GDC-0449 (40 mg/kg body weight; oral) or itraconazole (40 mg/kg body weight; oral) or sulindac (80 mg/kg body weight; topical in acetone), twice a week, 30 min prior to UVB irradiation. SSZ (300 ppm) was administered in drinking water [26]. These treatments and UVB-irradiation were carried out for 30 week. Additionally, in p50 decoy peptide experiment, 30 weeks following UVB irradiation, tumor lesions were treated with antennapedia control peptide or with p50 decoy peptide (Imgenex, San Diego, CA; intralesional; 25 μg/tumor in 50 μl PBS). The tumor number and size were recorded weekly using electronic Vernier Caliper as described earlier [86]. Data were presented as mean ± SE and plotted as a function of weeks on test. The experiment was terminated and all mice were euthanized as per IACUC recommendations. Skin and tumor tissues were harvested and processed for histological and biochemical analysis as described in the following sections.

### IR irradiation

IR irradiation was performed from X-RAD 320 Irradiation System (Precision X-ray, Inc. North Branford, CT) at the University of Alabama at Birmingham. Six to seven-week old Ptch1^+/−^/SKH-1 hairless mice were randomly divided into two groups of 20 mice each. Group-I mice served as age-matched control and left untreated (negative control). Group-II mice were irradiated with a single exposure of IR (5Gy) and kept for observations. The tumor number and size were recorded once a week using electronic Vernier Caliper. Data were presented as mean ± SE and plotted as a function of weeks on test. After termination of experiment, tumors were then harvested and analyzed for the biochemical, immunohistochemical and immunofluorescence analysis exactly as described earlier [26].

### β-gal staining and assessment of microscopic BCCs

For β-gal staining, 0.2% glutaraldehyde and 2% formalin-fixed tissues were treated with X-gal and iron buffer solution for overnight at room temperature and processed using the vender's protocol (Roche, Mannheim, Germany). Microscopic BCC-like lesions (β-gal-positive) were counted as numbers and size per unit area (mm^2^) of H&E and β-gal stained skin samples as described earlier [26].

### Scoring tumor incidence and multiplicity

Visible tumors greater than 2 mm in diameter were counted weekly and the data presented as % mice with tumors and also as number of tumors/mouse. Tumor size was recorded using electronic Vernier Caliper.

### Immunohistochemical staining

The paraffin tissue sections were deparaffinized, rehydrated and pretreated with 10 mM citrate buffer, pH 6.0 for 10 minutes at 95°C. The non-specific sites were blocked in 2.5% goat serum and bovine serum albumin (0.5% w/v) for 1 hour followed by overnight incubation at 4°C in the following primary antibodies: PCNA (Santa Cruz, sc-9857, 1:100), Gli-1 (Santa Cruz, sc-20687, 1:100); Hhip (Santa Cruz, sc-9406, 1:100). The sections were then washed and incubated with biotinylated secondary antibody. Color reaction was observed using the ABC peroxidase detection system using 3,3′-diaminobenzidine. Sections were counterstained with hematoxylin (Sigma), and mounted using permount (Sigma).

### Immunofluorescent staining

Frozen or paraffin tissue sections were processed for Immunofluorescent staining. Non-specific binding sites were blocked by 2.5% goat serum in bovine serum albumin (0.5% w/v) for 1 hour. The sections were then incubated with the following antibodies for overnight at 4°C: GR-1 (BD Biosciences, 1:100); CD11b (BD Biosciences, 1:100); CD11b (Abcam, 1:100); CD16 (Abcam, 1:100); CD11c (Abcam, 1:100); CD4 (R&D system, 1:50); CD4 (BD Biosciences, 1:100); MHCII (Abcam, 1:100); FOXP3 (Abcam, 1:100); Vitamin D receptor (VDR; Abcam, 1:100) Bcl-3 (Santa Cruz, sc-13038, 1:100); NFκB-p50 (Santa Cruz, sc-114, 1:100); NFκB-p52 (Santa Cruz, sc-298, 1:100); NFκB-p65 (Santa Cruz, sc-109, 1:100); Gli1 (Santa Cruz, sc-20687, 1:100); Cyclin D1 (Neomarkers, RM-9104-s1, 1:100); N-cadherin (Santa Cruz, sc-7939, 1:100) and K17 (Neomarkers, MS-489-S1, 1:100). After washing 3 times with PBS, the sections were incubated with Alexa Fluor^®^ 488 or Alexa Fluor^®^ 594 secondary antibodies (Life Technologies) for 1 hour at room temperature. After removal of antibodies, the sections were rinsed with PBS and mounted with mounting medium containing DAPI. Fluorescence was immediately recorded on an Olympus EX51 microscope.

### Immunoblotting

100 μg of total protein was separated on a 10% SDS-PAGE gel and blotted onto nitrocellulose membrane. After transfer, nonspecific sites were blocked with 5% (W/V) nonfat-dry milk in TTBS (0.1% Tween-20, 20 mM Tris base, 137 mM NaCl, 3.8 mM HCl, pH 7.6) for 2 hours at ambient temperature followed by overnight probing with primary antibody at 4°C. After washing the blot three times in TTBS for 10 minutes each, the membrane was incubated for 1 hour with horseradish peroxidase-conjugated secondary antibody (dilution- 1:2,000, Thermo Scientific, Rockford, IL). The blot was washed three times in TTBS for 10 minutes each and was developed with ECL according to the manufacturer's instructions (Amersham, Arlington Heights, IL).

### Terminal deoxynucleotidyl transferase–mediated nick end labeling (TUNEL) assay

TUNEL assay was done by using *in situ* cell death detection, fluorescein kit from Roche Applied Science following manufacturer's guidelines.

### MPO activity

MPO activity in the skin samples was determined by using Myeloperoxidase Peroxidation Assay Kit (Cayman Chemical Company, Ann Arbor, Michigan) according to manufacturer protocol.

### Immunoprecipitation

Tissue lysate (200 μg total protein) was incubated for 2 hours at 4°C with antibody (1 μg) followed by incubation with protein A-Sepharose (Sigma, cat: P9424) for 1 h at 4°C. The immunoprecipitates were washed five times with tissue lysis buffer containing 10 mM Tris pH 7.4, 1.0% Triton X-100, 0.5% NP-40, 150 mM NaCl, 20 mM NaF, 0.2 mM sodium orthovanadate, 1 mM EDTA, 1 mM EGTA, 0.2 mM PMSF and analyzed by western blotting as described above.

### RNA extraction & RT-PCR

Total RNA isolation from skin and tumor was carried out using Purezol (Biorad) following the manufacturer's instruction. 1 μg of total RNA was reverse-transcribed into cDNA using iScript Synthesis System (Biorad) for RT-PCR. The primers used were as follows: TNFα, forward TGCCTATGTCTCAGCCTCTT and reverse ACTTGGTGGTTTGCTACGAC; IL1β, forward GATAACCTGCTGGTGTGTGAC and reverse TGAGG TGCTGATGTACCAGTT; MnSOD, forward GACCTG CCTTACGACTATGG and reverse GACCTTGCTCCTT ATTGAAGC; TRAIL, forward TCACCAACGAGATGA AGCAGC and reverse CTCACCTTGTCCTTTGAGACC; Cyclin D1, forward CTCTGGCTCTGTGCCTTTCT and reverse CCGGAGGACTCAGAGCAAATC; Cyclin D2, forward CCTCACGACTTCATTGAGCA and reverse ATGCTGCTCTTGACGGAACT; Cyclin D3, forward GCATACTGGATGCTGGAGGT and reverse ACAGAGG GCCAAAAAGGTCT; Cyclin E, forward CTTTCAGTC CGCTCCAGAAA and reverse AGCCAATCCAGAA GAACTGC; Ptch1, forward AACAAAAATTCAACC AAACCTC and reverse TGTCTTCATTCCAGTTGA TGTG; Ptch2, forward TGCCTCTCTGGAGGGCTTCC and reverse CAGTTCCTCCTGCCAGTGCA; Gli1, forward GTCGGAAGTCCTATTCACGC and reverse CAGTCTGCTCTCTTCCCTGC; Gli2, forward GAGCA GAAGCCCTTCAAG and reverse GACAGTCTTCACA TGCTT; Gli3, forward CAAGCCTGATGAAGACCTCC and reverse GCTTTGAACGGTTTCTGCTC.

### Inflammation cytokines/chemokines PCR array

PCR Array was done using SABiosciences PCR Array System as described earlier [117]. Briefly, cDNA synthesis was done using RT^2^ First Strand kit. Real-time PCR was done with Mouse Inflammatory Cytokines & Receptors PCR Arrays using RT2 qPCR Master Mix. For each group, 3 skin samples were used for PCR array analysis. Relative fold changes of gene expression were calculated according to the manufacturer's instruction and software.

### DNA extraction and mutation screening for the Ptch1 gene

Tissues were then digested in a buffer with proteinase K (Qiagen) at 55°C overnight. The tumor genomic DNA was then extracted following the instructions. A set of primers (Ptch1 Wild-type, forward CTGCGGCAAGTTTTTGGTTG and reverse AGGGCTTCTCGTTGGCTACAAG; Ptch1 mutant, forward GCCCTGAATGAACTGCAGGACG and reverse CACGGGTAGCCAACGCTATGTC flanking exon 23 of the Ptch1 gene were used to amplify tumor genomic DNA. These PCR amplicons were then purified and sequenced by GeneWiz Inc. The mutation was identified by visual inspection and comparison with control sequences generated from wild-type samples.

### EMSA

NFκB consensus oligonucleotide (5′-AGT TGA GGG GAC TTT CCCAGG C-3′, 3′-CTA GCT TGA CTG GCG GGC GCC GGG CA-5′) was purchased from Promega (E3292; Promega Corp., Madison, WI). 3.5 pmol of NFκB consensus oligonucleotide was 5′-end-labeled with [γ-^32^P] ATP using T4 polynucleotide kinase (Promega Corp., Madison, WI). Nuclear protein extracts (10 μg) were incubated on ice for 10 min. with 4 μl of gel shift binding 5X buffer (Promega Corp. Madison, WI) containing 20% glycerol (v/v), 5 mM MgCl_2_, 2.5 mM EDTA, 2.5 mM DTT, 250 mM NaCl, 50 mM Tris-HCl (pH 7.5), 1μl of γ-^32^P labeled oligonucleotide was then added, and incubation was continued for 20 min at room temperature. In the competition experiments, a 100-fold molar excess of unlabeled probe was added before the labeled probe. Reaction products were separated on 4% polyacrylamide gels run in 0.5XTBE buffer at 120V at room temperature. After electrophoresis, the gels were dried and exposed to X-ray film overnight at −70°C. In the supershift assays, 2 μg of affinity-purified polyclonal antibodies were added after binding reactions and incubation was further continued for 30 min at room temperature. The antibodies for the supershift assays were purchased from Santa Cruz (NFκB p65, sc-109; NFκB p50, sc-114; NFκB p52, sc-298).

### IPTG-mediated knock-down of Bcl3

We used the pLKO_IPTG_3XLacO vector (Sigma), which has been designed to contain a LacI (repressor) and a modified human U6 shRNA promoter with LacO (operator) sequences, to mediate inducible Bcl3 specific RNA interference in basal cell carcinoma cells (ASZ cells). In the absence of IPTG (isopropyl-β-D-thiogalactoside), an analogue of lactose, LacI binds to LacO preventing expression of the Bcl3 shRNA. When IPTG is present, the allosteric LacI repressor changes conformation, releasing itself from lacO modified human U6 promoter, and subsequently allows expression of the Bcl3 shRNA. Stable pools and single cell clones was propagated at the appropriate puromycin concentration without the addition of IPTG. A 70–80% knock down efficiency was observed after 72 hours treatment of IPTG.

## SUPPLEMENTARY FIGURES


