# Supplementary material for: Shh and p50/Bcl3 signaling crosstalk drives pathogenesis of BCCs in gorlin syndrome
Source: Oncotarget. 2015 Sep 15;6(34):36789–814. doi: 10.18632/oncotarget.5103 (PMC4742211; doi:10.18632/oncotarget.5103)
Supplement: Supplementary file 1 [file oncotarget-06-36789-s001.pdf]

SUPPLEMENTARY FIGURES

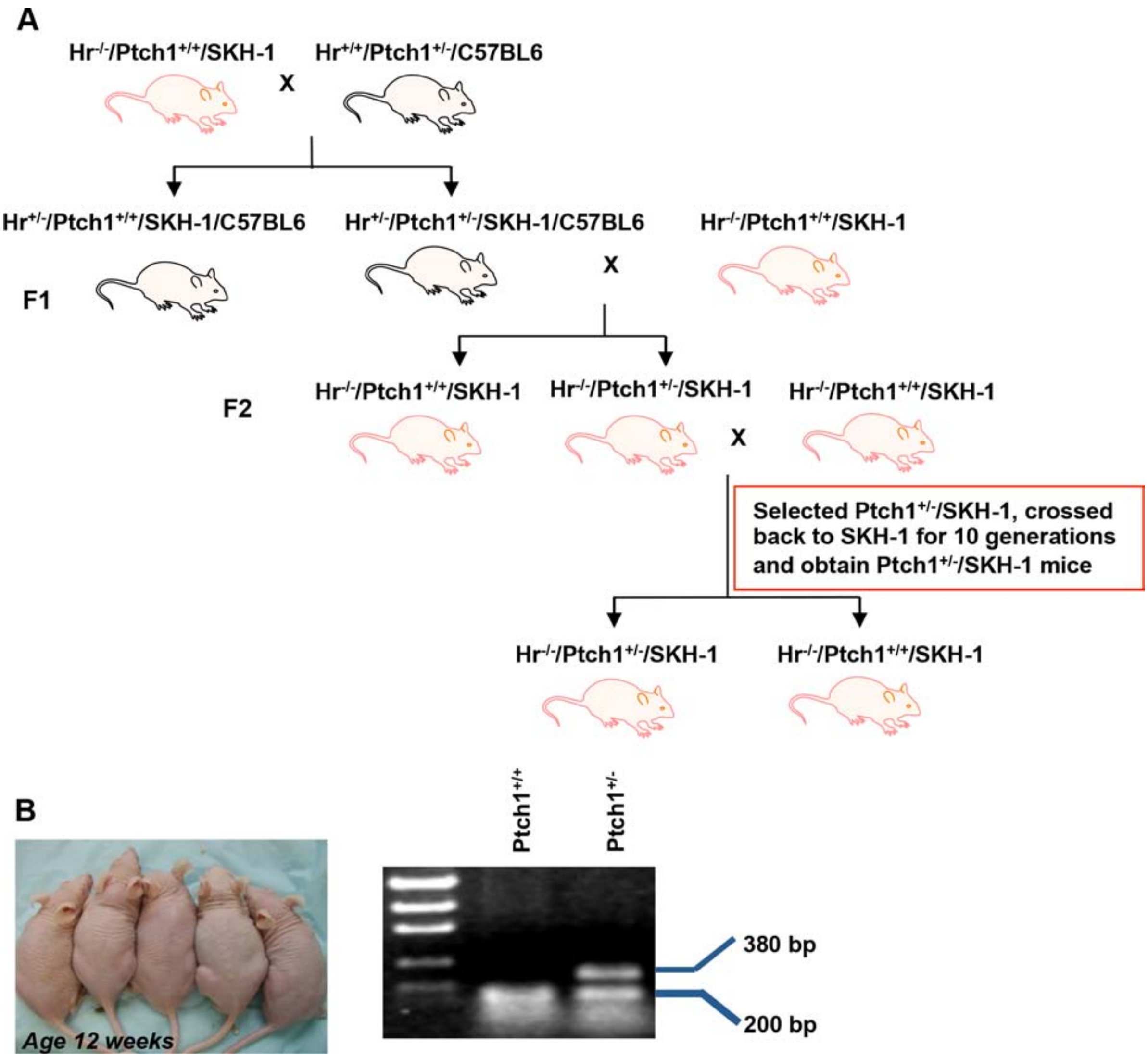

Supplementary Figure S1: Breeding scheme for the generation of Ptch1 heterozygous (Ptch1+/−) SKH-1 mice. A. Male Ptch1$^{+/-}$/C57BL/6 mice were crossed with female SKH-1 hairless mice to obtain Ptch1$^{+/-}$/SKH-1/C57BL/6 mice. The male littermates of Ptch1$^{+/-}$/SKH-1/C57BL/6 were further bred with female SKH-1 animals for more than 10 generations to generate Ptch1$^{+/-}$/SKH-1 mice of hairless genetic background. **B.** Tail skin DNA was subjected to PCR analysis for genotyping of these animals.

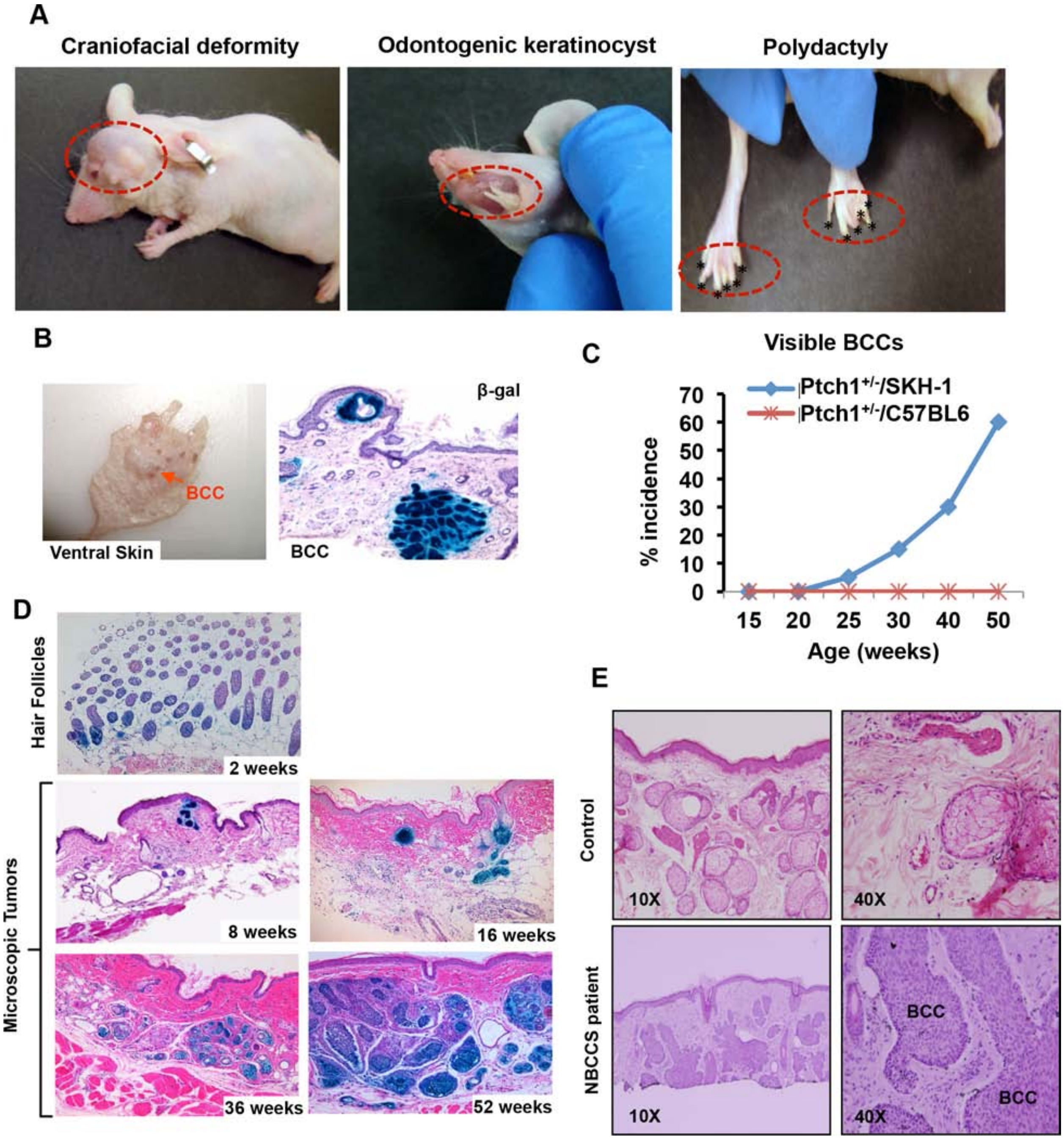

Supplementary Figure S2: Phenotypic abnormalities, and development of spontaneous macroscopic and microscopic BCCs in Ptch1$^{+/-}$/SKH-1 mice. A. Phenotypic abnormalities observed in Ptch1$^{+/-}$/SKH-1 mice showing, craniofacial abnormalities, odontogenic keratocyst and polydactyly. **B.** Spontaneously BCCs on ventral skin of Ptch1$^{+/-}$/SKH-1 mouse. **C.** Percentage incidence of spontaneous BCCs in Ptch1$^{+/-}$/SKH-1 mice and **D.** β-gal staining showing growth of spontaneous microscopic BCCs in different age groups. **E.** H&E staining showing presence of BCCs in patients with NBCCS.

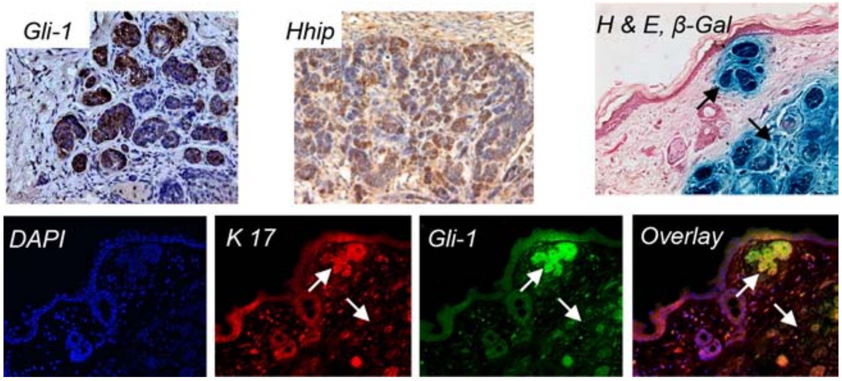

Supplementary Figure S3: Immunostaining showing expression of BCC biomarkers Gli-1, Hhip and co-expression of K17 and Gli-1 in spontaneous BCCs in Ptch1$^{+/-}$/SKH-1 mice.

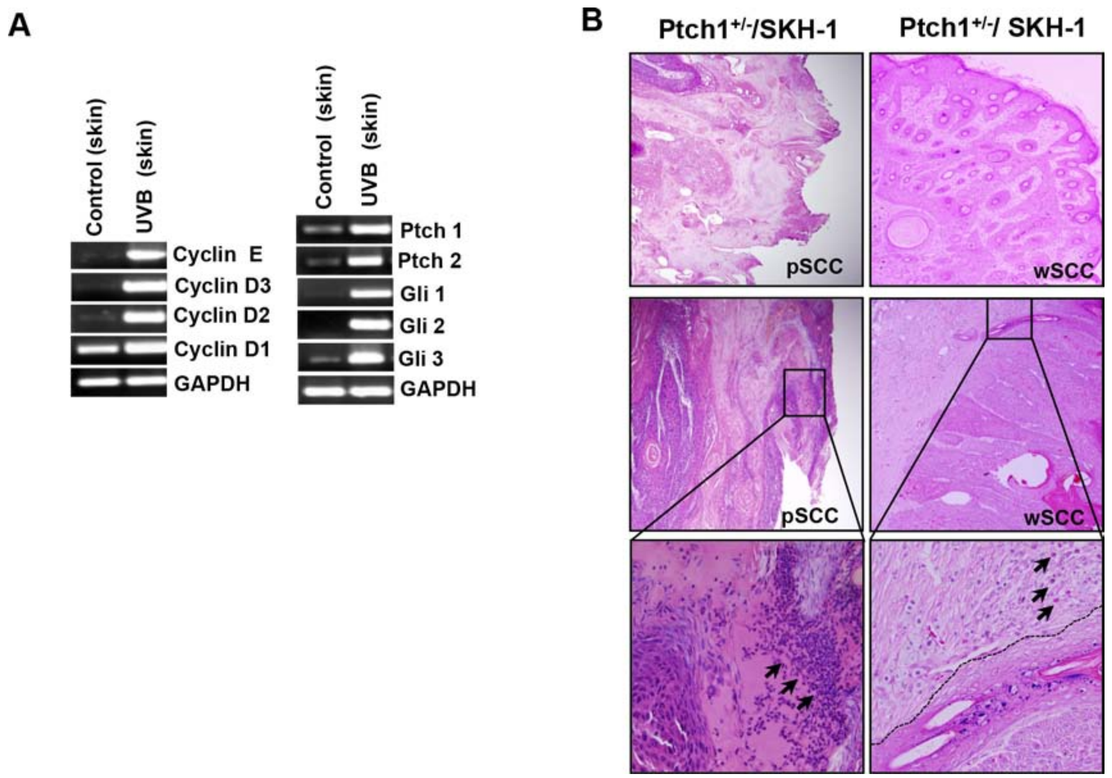

Supplementary Figure S4: Up-regulation of cell cycle-regulatory proteins and Shh signaling molecules in tumor-adjacent skin of UVB-irradiated Ptch1$^{+/-}$/SKH-1 mice. A. Semi-quantitative PCR analysis showing expression of cell cycle and Shh regulatory components in the skin of Ptch1$^{+/-}$/SKH-1 mice; **B.** Representative histology of identical tumors in Ptch1$^{+/-}$/SKH-1 and Ptch1$^{+/+}$/SKH-1 mice showing differential infiltration of inflammatory cells, and well-differentiated vs poorly-differentiated SCCs development (original magnification 10x). pSCC, poorly-differentiated SCC; wSCC, well-differentiated SCC.

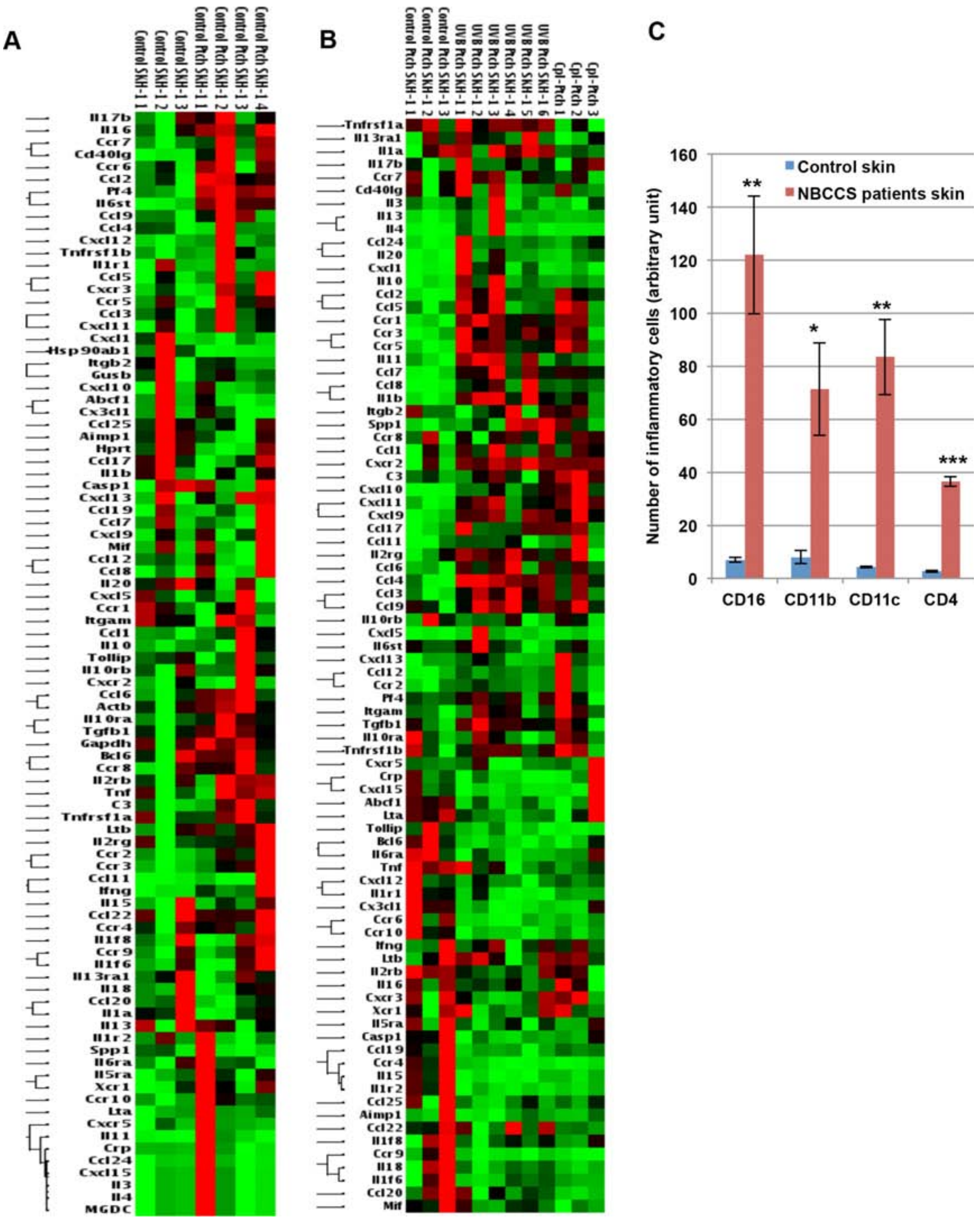

Supplementary Figure S5: Heat map showing differential expression of inflammatory cytokines/ chemokines in the skin of Ptch1$^{+/+}$/SKH-1 and Ptch1$^{+/-}$/SKH-1 mice and microenvironment of BCCs from patients with NBCCS. A. Baseline expression of inflammatory cytokines/chemokines genes in the skin of Ptch1$^{+/+}$/SKH-1 and Ptch1$^{+/-}$/SKH-1 mice at the age of 30 weeks; **B.** expression of inflammatory cytokines/chemokines genes in the skin of age-matched controls, UVB-irradiated and cyclopamine+UVB-treated Ptch1$^{+/-}$/SKH-1 mice. **C.** Number of CD11b, CD16, CD11c and CD4-positve inflammatory cells in tumor microenvironment of skin of patients with NBCCS syndrome compared to normal human control skin. *P* values *<0.05, **<0.01, ***<0.001 show level of significance.

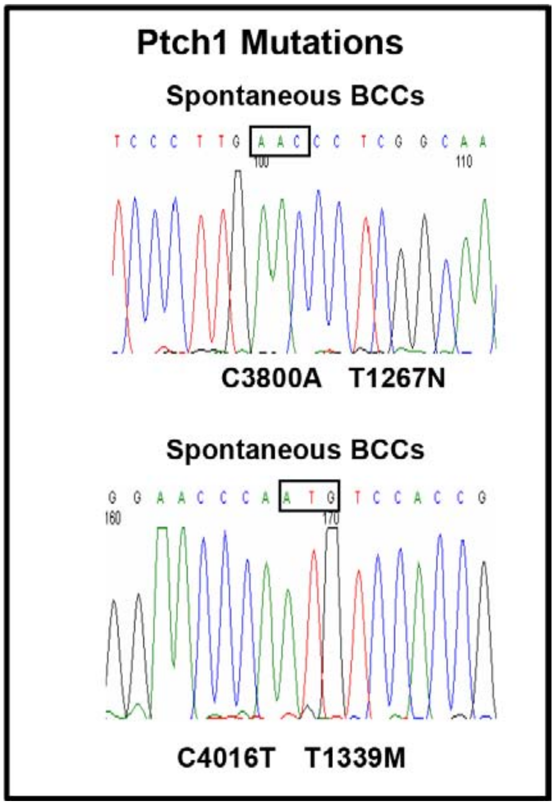

Supplementary Figure S6: Ptch1 gene mutations in spontaneous BCCs in Ptch1$^{+/-}$/SKH-1 mice.

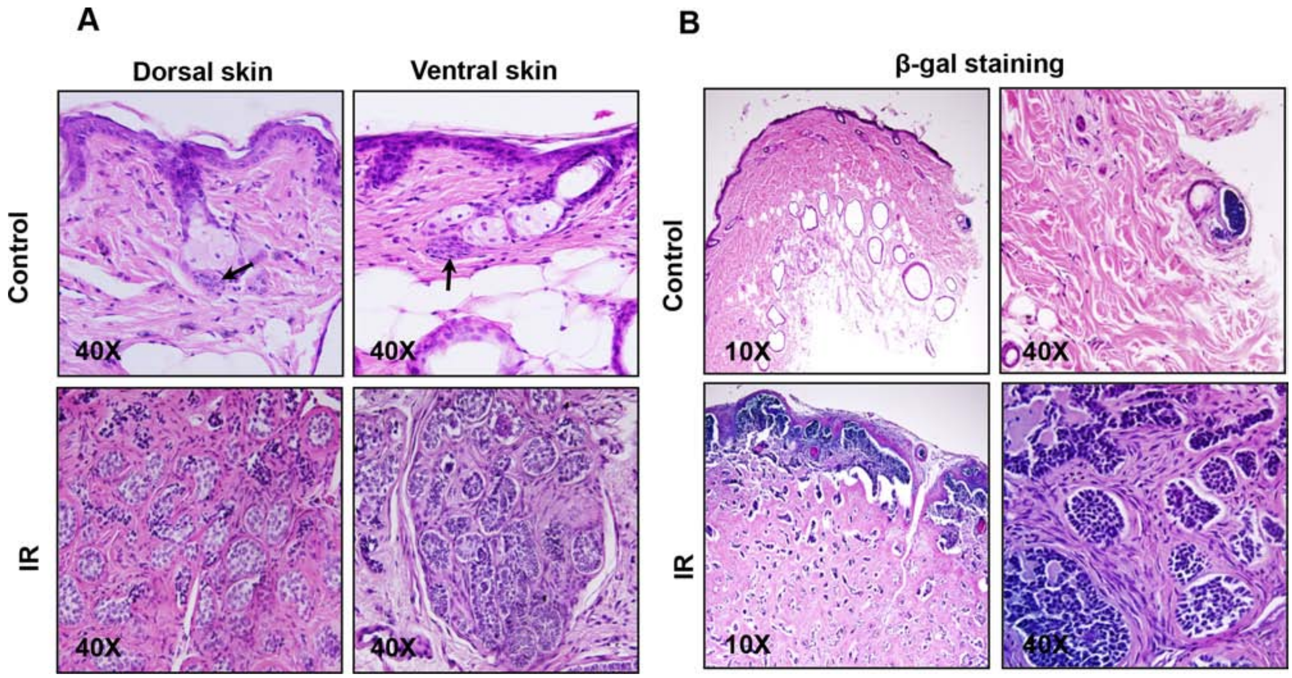

Supplementary Figure S7: H&E and β-gal staining of microscopic BCCs induced in IR-irradiated Ptch1$^{+/-}$/SKH-1 mice. Ptch1$^{+/-}$/SKH-1 mice were irradiated with a single dose of IR (5 Gy). IR irradiation induces multiple of BCCs on both dorsal and ventral skin. **A.** H&E staining and **B.** β-gal staining of IR-induced BCCs in Ptch1$^{+/-}$/SKH-1 mice.

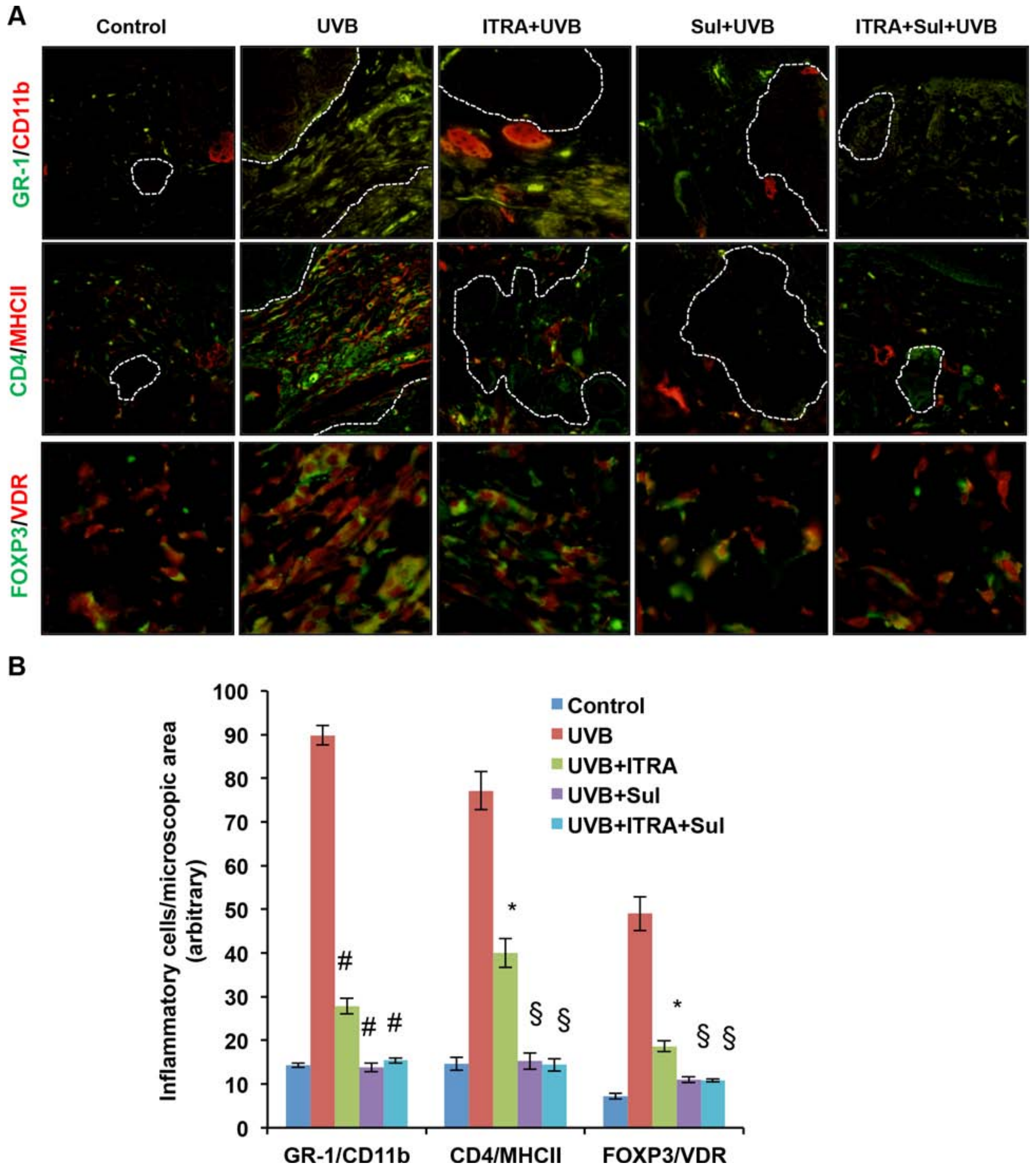

Supplementary Figure S8: Effects of ITRA and Sul alone and in combination on infiltrating inflammatory cells in the skin and in the microenvironment of UVB-induced BCCs in Ptch1$^{+/-}$/SKH-1 mice. Orally administered ITRA (40 mg/kg body weight) or topically administered Sul (80mg/kg body weight) alone or in combination were administered to UVB -irradiated (180 mJ/cm^2) Ptch$^{+/-}$/SKH-1 mice, 30 min prior to irradiation for 30 weeks. Panel **A. B.** represent immunofluorescence staining (without DAPI) of GR-1 (green)/CD11b (red)-positive myeloid cells, CD4 (green)/MHCII (red)-positive T helper cells and FOXP3 (green)/ VDR (red)-positive regulatory T cells. *P* value *<0.05, §<0.01, #<0.001

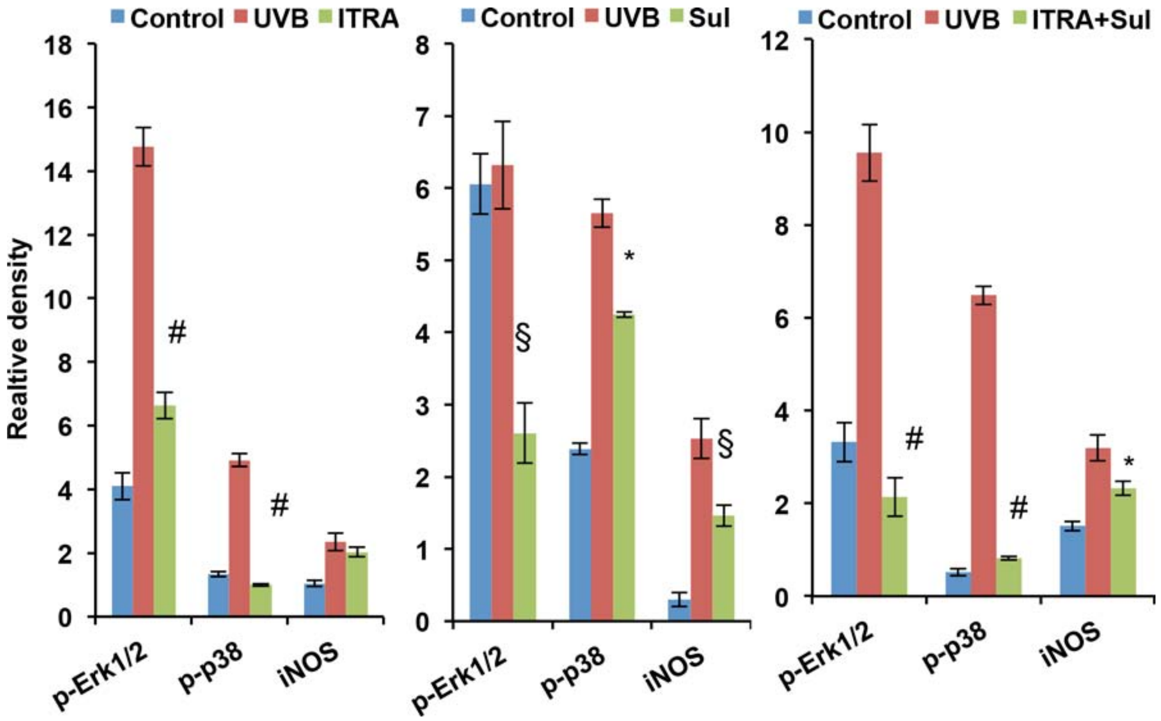

Supplementary Figure S9: Effects of ITRA and Sul alone and in combination on inflammatory and proliferating biomarkers in skin of UVB-irradiated Ptch1$^{+/-}$/SKH-1 mice. Densitometric analysis of Erk1/2, p38, and iNOS expression in UVB-induced skin lesions in Ptch1$^{+/-}$/SKH-1 mice. The combination of ITRA and Sul significantly diminished expression of these markers. *P* value *<0.05, §<0.01, #<0.001.

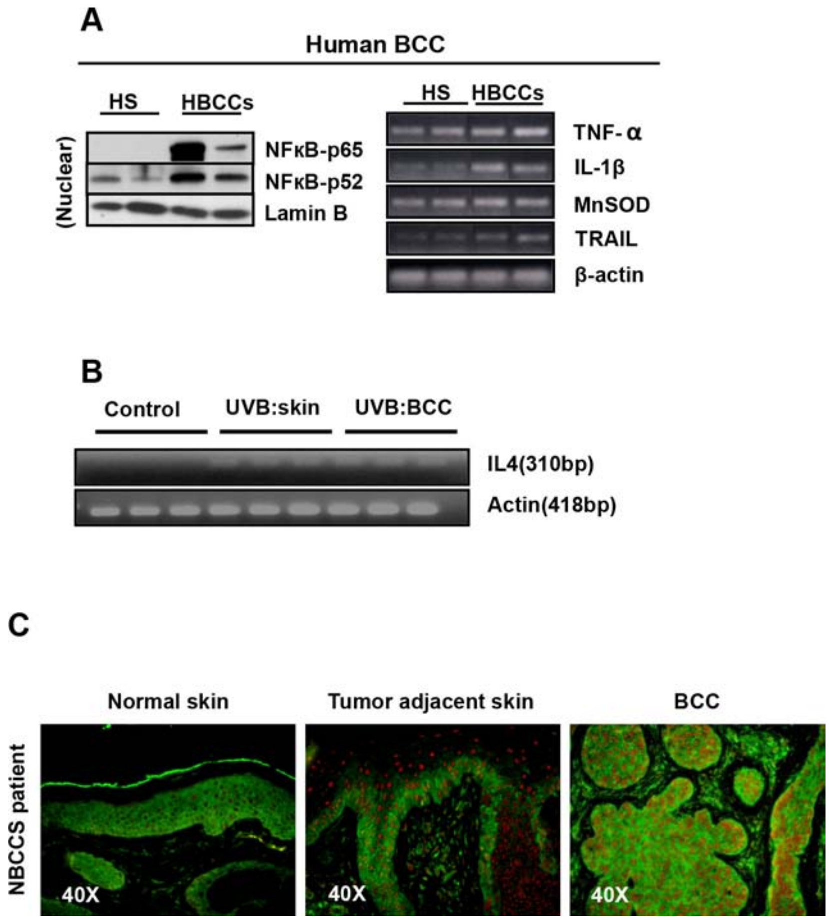

Supplementary Figure S10: Expression of NFκB proteins and transcriptional targets of NFκB in human BCCs and murine BCCs. A. Western blot analysis and semi-quantitative RT-PCR analysis of NFκB (NFκB-p65, NFκB-p52) and its transcriptional target genes TNF-α, IL-1β, MnSOD, and TRAIL in human BCCs (NE = nuclear extracts); **B.** Semi-quantitative RT-PCR analysis showing up-regulation of mRNA levels of IL4 in UVB-induced BCCs and tumor adjacent skin of $Ptch^{+/-}$/SKH-1 mice; **C.** Co-expression of Bcl3 and p50 in patients with NBCCS samples showing nuclear localization of these proteins.

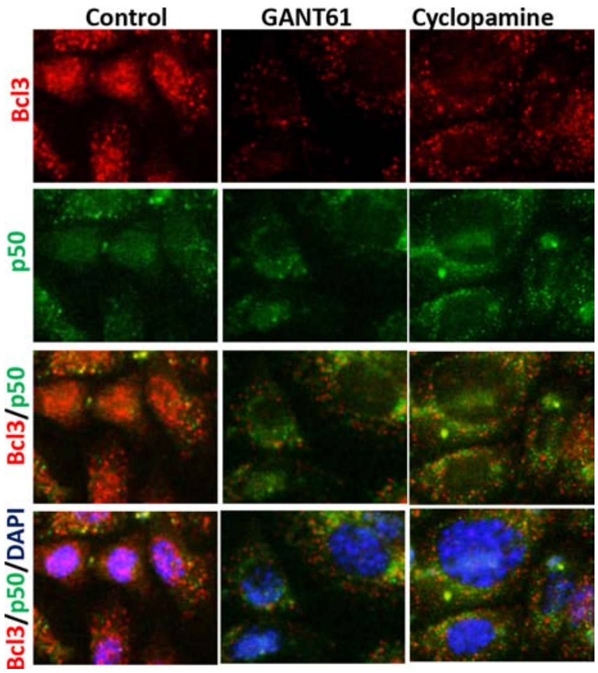

Supplementary Figure S11: Shh inhibitors reduced the nuclear translocation of Bcl3/p50 complex in ASZ001 cell line. ASZ cells were treated with GANT61 (10 μM) and cyclopamine (5 μM) in 154CF media supplemented with human keartinocyte and $CaCl_2$ at 37°C for 24 h. ASZ001 cells were immunostained with Bcl3 and p50 antibodies.

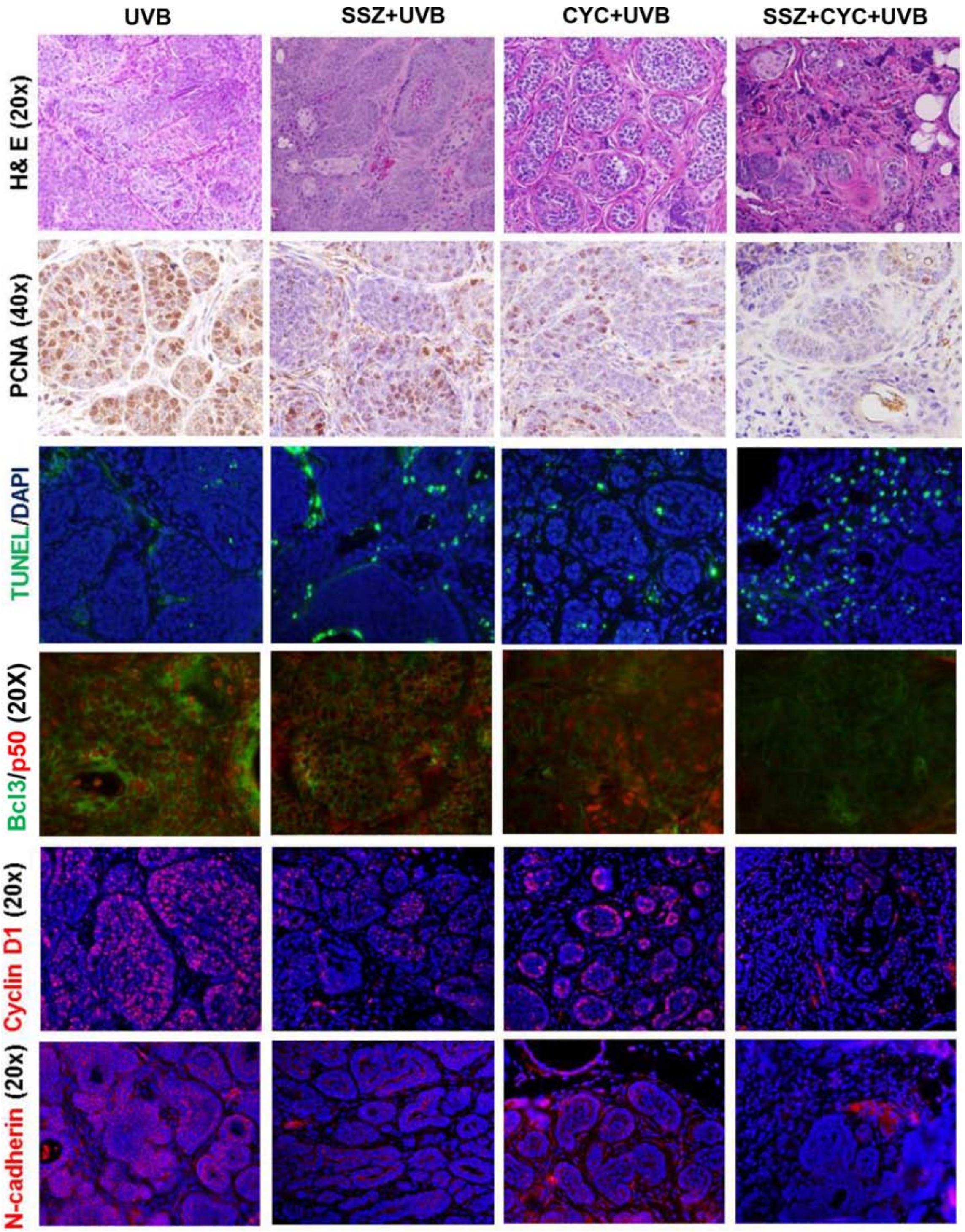

Supplementary Figure S12: Effects of sulfasalazine (SSZ) and cyclopamine (CYC) alone and in combination on PCNA, apoptosis, Bcl3, p50, Cyclin D1 and N-cadherin in the UVB-induced BCCs in Ptch1$^{+/-}$/SKH-1 mice. Orally administered SSZ (300 ppm in drinking water) or i.p. administered CYC (20mg/kg body weight) alone or in combination were administered to UVB -irradiated (180 mJ/cm^2) Ptch1$^{+/-}$/SKH-1 mice, 30 min prior to irradiation for 30 weeks. Panel I represents H&E staining; panel II represents immunohistochemical staining of PCNA; panel III represents TUNEL-positive cells; panel IV showing immunofluorescence staining of Bcl3 (red) /NFκBp50 (green)-positive BCC cells; panel V showing immunofluorescence staining of cyclin D1 (red) with DAPI (blue); panel VI showing immunofluorescence staining of N-Cadherin (red) with DAPI (blue).

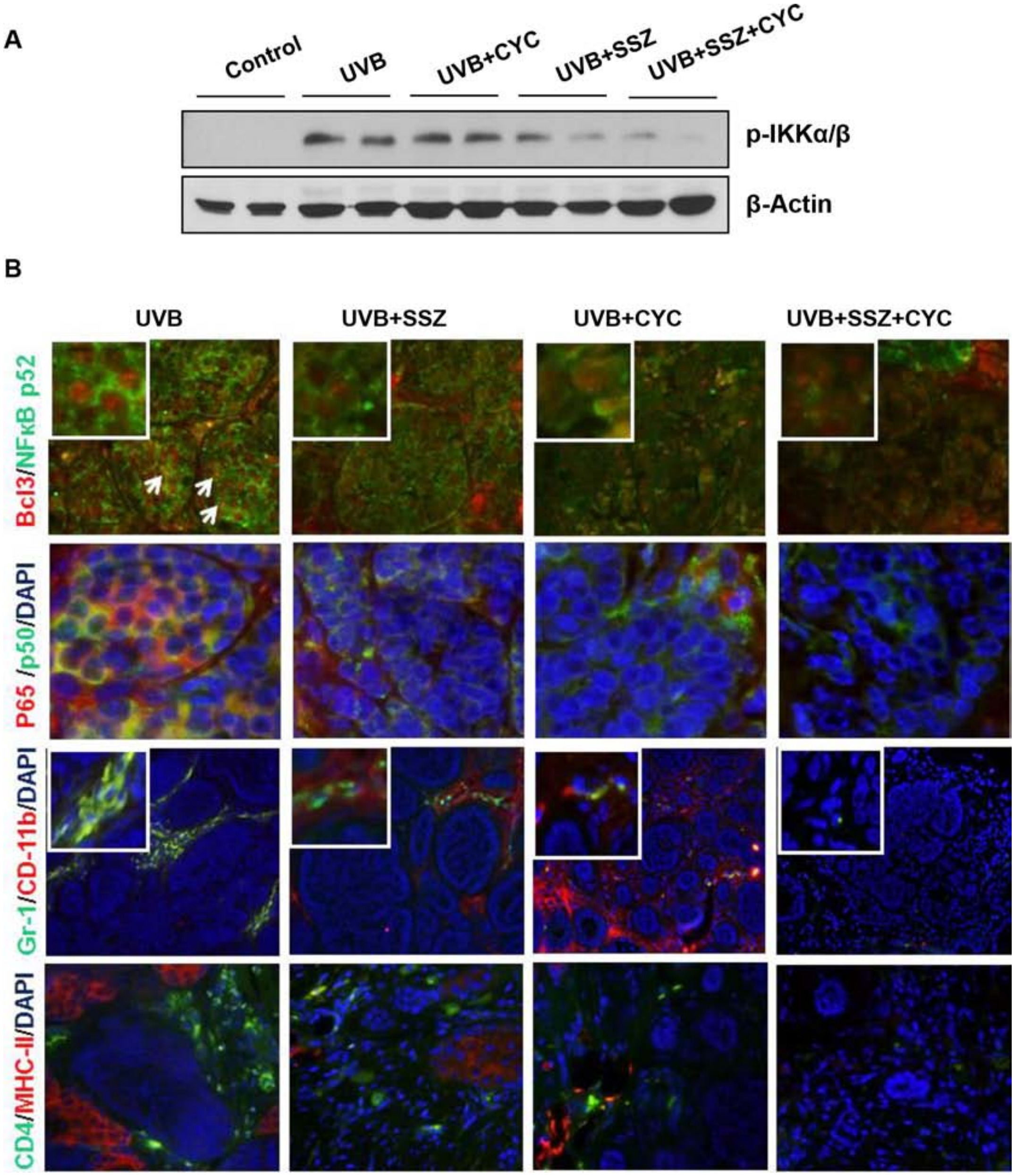

Supplementary Figure S13: Effects of sulfasalazine (SSZ) and cyclopamine (CYC) alone and in combination on expression of Bcl3/p52, p65/p50 and on infiltrating inflammatory cells in the skin and in the microenvironment of UVB-induced BCCs in Ptch1$^{+/-}$/SKH-1 mice. Orally administered SSZ (300 ppm in drinking water) or i.p. administered CYC (20 mg/kg body weight) alone or in combination were administered to UVB -irradiated (180 mJ/cm^2) Ptch1$^{+/-}$/SKH-1 mice, 30 min prior to irradiation for 30 weeks. **A.** Western blot analysis of p-IKKα/β in skin tissues treated with SSZ and CYC alone and in combination. Panel I represents immunofluorescence staining of Bcl3 (red) and NFkBp52 (green); panel II represents immunofluorescence staining of p65 (red), p50 (green) and DAPI (blue); panel III represent immunofluorescence staining GR-1 (green)/CD11b (red)-positive myeloid cells, CD4 (green)/MHCII (red)-positive T helper cells.
